# Metals in Imaging of Alzheimer’s Disease

**DOI:** 10.3390/ijms21239190

**Published:** 2020-12-02

**Authors:** Olga Krasnovskaya, Daniil Spector, Alexander Zlobin, Kirill Pavlov, Peter Gorelkin, Alexander Erofeev, Elena Beloglazkina, Alexander Majouga

**Affiliations:** 1 Chemistry Department, Lomonosov Moscow State University, Leninskie Gory 1,3, 119991 Moscow, Russia; zlobinabchem@gmail.com (A.Z.); kirill.pavlov2011@mail.ru (K.P.); peter.gorelkin@gmail.com (P.G.); Erofeev@polly.phys.msu.ru (A.E.); beloglazki@mail.ru (E.B.); alexander.majouga@gmail.com (A.M.); 2 Department of Materials Science of Semiconductors and Dielectrics, National University of Science and Technology (MISIS), Leninskiy Prospect 4, 101000 Moscow, Russia; 3 Mendeleev University of Chemical Technology of Russia, Miusskaya Ploshchad’ 9, 125047 Moscow, Russia

**Keywords:** Alzheimer disease, amyloid, PET, SPECT, MRI

## Abstract

One of the hallmarks of Alzheimer’s disease (AD) is the deposition of amyloid plaques in the brain parenchyma, which occurs 7–15 years before the onset of cognitive symptoms of the pathology. Timely diagnostics of amyloid formations allows identifying AD at an early stage and initiating inhibitor therapy, delaying the progression of the disease. However, clinically used radiopharmaceuticals based on ^11^C and ^18^F are synchrotron-dependent and short-lived. The design of new metal-containing radiopharmaceuticals for AD visualization is of interest. The development of coordination compounds capable of effectively crossing the blood-brain barrier (BBB) requires careful selection of a ligand moiety, a metal chelating scaffold, and a metal cation, defining the method of supposed Aβ visualization. In this review, we have summarized metal-containing drugs for positron emission tomography (PET), magnetic resonance imaging (MRI), and single-photon emission computed tomography (SPECT) imaging of Alzheimer’s disease. The obtained data allow assessing the structure-ability to cross the BBB ratio.

## 1. Introduction

Alzheimer’s disease is the most common form of neurodegenerative disease. This pathology is characterized by the presence of extracellular amyloid plaques and intracellular neurofibrillary tangles (NFTs) in the brain [1]. One of the hallmarks is the extracellular amyloid plaques in aggregated forms of a peptide called amyloid-β (Aβ), appearing years before the onset of symptoms [2,3,4,5].

**Figure 1:**
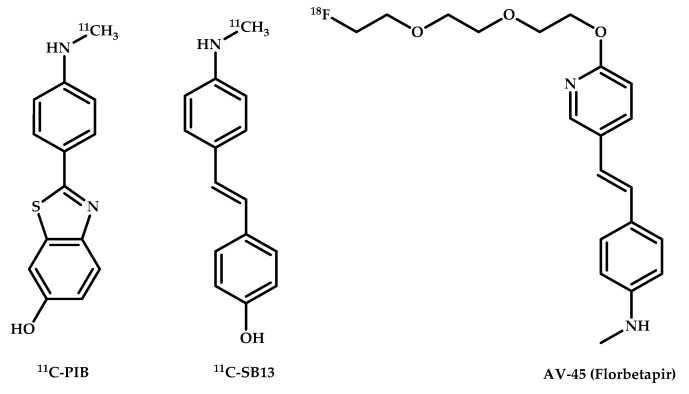
Pittsburgh compound B (PiB), [11C]4-N-Methylamino-4′-hydroxystilbene (SB-13), and Florbetair (AV-45), AD PET imaging agents.

Timely diagnostic imaging plays an important role in managing AD. Several positron emission tomography (PET) imaging agents have been developed that bind to different amyloids, such as 2-(1,1-dicyanopropen-2-yl)-6-(2-[18F]-fluoroethyl)-methylamino-naphthalene [^18^F]FDDNP, [^11^C]Pittsburgh Compound-B (PiB), [^18^F]Florbetapir, [^18^F]Florbetaben, and [^18^F]Flutemetamol, allow obtaining semiquantitative information about amyloid deposition in patients, which allows presaging the development of clinical symptoms of AD 7–15 years before their occurrence [6,7,8,9,10] (Figure 1). But using these drugs requires an expensive laborious synthesis with confirmation of radio purity at each stage. The short half-lives of the currently used radionuclides ^11^C (20.4 min) and ^18^F (109.8 min) may also limit the widespread use of these imaging agents [11,12].

Although metal cations such as Cu(II), Zn(II), and Fe(III) proved to coordinate undesirably with histidine residues at the N-terminus of Aβ, promoting Aβ aggregation and stabilization of Aβ oligomers [13], an increased accumulation of these metals in Aβ-amyloids raises the possibility of designing Cu(II)-, Zn(II)-, and Fe(III)-based metal complexes for the diagnosis and theranostics of AD. AD diagnostic agents radiolabeled with ^64^Cu are attractive not only due to the simple and fast introduction of radionuclide at the last stage of non-radioactive synthesis, but also due to the 12.7 h half-life of ^64^Cu radionuclide, ideal for PET imaging [14].

Another promising PET radionuclide is ^68^Ga. Positron-emitting ^68^Ga can be obtained from a ^68^Ge/^68^Ga generator, which would allow a cyclotron-independent distribution of PET. The parent nuclide, ^68^Ge, has a half-life of 271 days, and the generators can provide sufficient quantities of ^68^Ga for up to one year, resulting in a relatively inexpensive and reliable source of a positron-emitting radionuclide [15,16].

In addition to PET imaging of amyloids, single-photon emission computed tomography (SPECT) and magnetic resonance imaging (MRI) are alternative diagnostic tools for AD visualization, able to overcome the limitations of PET imaging in terms of cost and broad accessibility [17]. The technetium-99 m (^99m^Tc) radioisotope for SPECT imaging can be cyclotron-independently prepared by a ^99^Mo/^99m^Tc generator [18]. The MRI imaging allows nonradioactive diagnostics and is also cheaper and faster than PET imaging. The Gd^3+^ PET imaging agents for Aβ visualization are also of interest [19].

The development of effective diagnostic and therapeutic agents targeting amyloid is not a trivial task. The blood-brain barrier (BBB) is a highly selective, semipermeable barrier, consistent of cerebrovascular endothelial cells, surrounded by extracellular matrix, astrocytes, and pericytes [20], which prevents potential therapeutics from reaching the cerebral target, thus limiting their efficacy [21]. Various approaches to effective brain delivery are developed, such as chemical drug delivery systems [22], e.g., a drug conjugation with dihydropyridine, mannitol, or aromatic substances [23], physical methods, such as focused ultrasound [24] or sonophoresis [25], and biological methods, e.g., drug conjugation with polycationic proteins or amino acids [26].

The complexity of the architecture of the blood-brain barrier, as well as the significant difficulties accompanying the development of drugs capable of overcoming it, prompts the creation of in vitro models of the BBB, such as microfluidic models [27], brain organoids [28], and microvascular systems [29].

The BBB permeability of a compound is related to its lipophilicity, expressed by the water/octanol partition coefficient, log P_oct/water_, molecular weight (MW), and plasma pharmacokinetics [30]. Low-MW amphiphilic molecules with log P_oct/water_ ≈ 2 have optimal BBB penetration [31]. Conjugating an Aβ-affinity moiety, a metal-chelating moiety, and a metal cation in one scaffold is often difficult, and the resulting drugs are often unable to cross the BBB.

Sedgwick et al. summarized metal-based imaging agents for neurodegenerative disease diagnostics [32]. Gomes et al. also summarized an interaction of metal complexes with the Aβ peptide [33]. Liu et al. reported potential applications of metal-based agents in therapy, diagnosis, and theranosis of AD [34].

In this review, we summarize various solutions in the design of amyloid-affinity drugs capable of effectively crossing the BBB, and different approaches for designing Aβ-affinity drugs for diagnosing AD. Three summary tables can be conveniently used to evaluate the structure of the ligand and the result of brain penetration by the coordination compound based on it, noting the successful and unsuccessful attempts to create drugs for diagnosing AD. This review will be useful to researchers for developing approaches for designing Aβ-affinity drugs for both the therapy and diagnostics of AD.

## 2. Copper Coordination Compounds for PET Imaging of Alzheimer Disease

PET diagnostics is based on registering a pair of gamma quanta resulting from the annihilation of electrons and positrons that arise during the positron-beta decay of a radionuclide. Annihilation of the positron, which remained in the tissue, with one of the electrons of the medium, generates two gamma quanta with the same energy, scattering in opposite directions along one straight line. A set of detectors makes it possible to obtain a three-dimensional reconstruction of the distribution of the radionuclide in the body tissue [35].

The radionuclide ^64^Cu has a long half-life (t_1/2_ = 12.7 h, β^+^ = 17%, β^−^ = 39%, e-capture decay EC = 43%, E_max_ = 0.656 MeV) and can be considered an ideal PET tracer [36]. Copper-coordination compounds are promising for PET diagnostics of AD because of not only the emission properties but also the increased affinity of amyloids for copper cations, which would further increase the accumulation of copper-containing drugs in the therapeutic target [37]. 

A standard approach in developing Aβ PET imaging drugs is a conjugation of an Aβ-binding benzothiazole, benzofuran, or stilbene scaffold, with a metal-chelating moiety. Thiosemicarbazone derivatives are often used as a metal-chelating agent, based on the diacetylbis(N(4)-methylthiosemicarbazonato Cu-ATSM drug [38].

**Figure 2:**
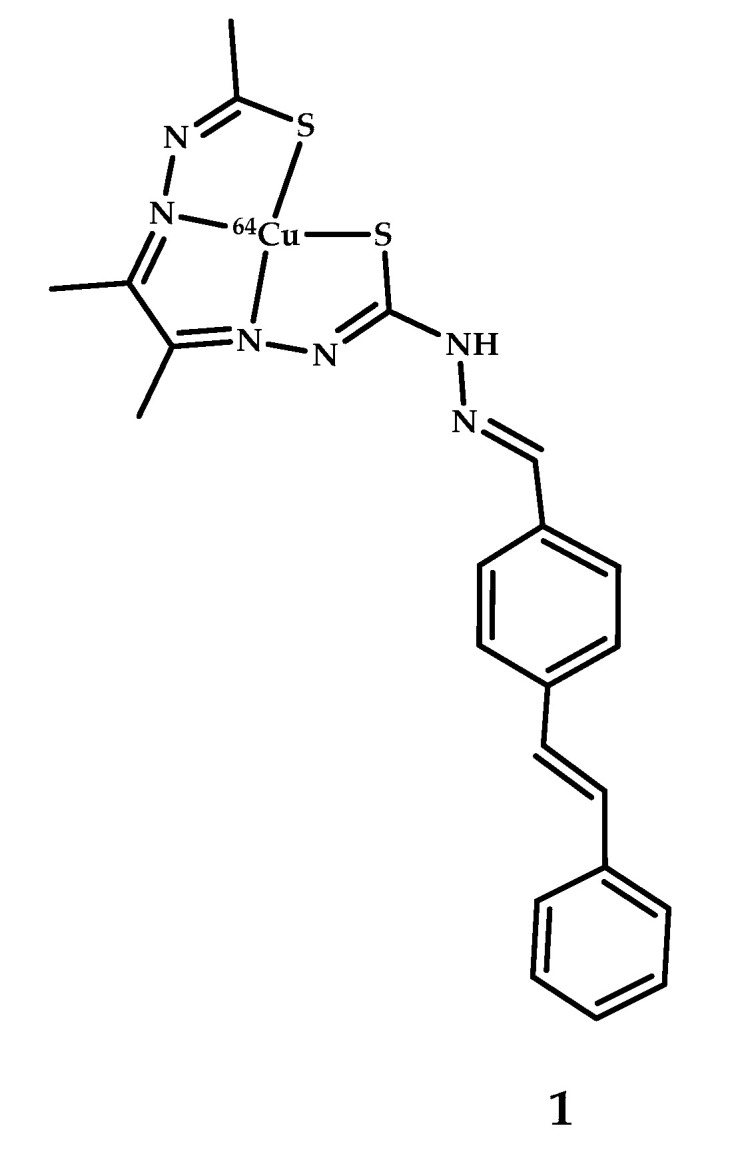
^64^Cu(II)-ATSM derivative **1** conjugated with stilbene functional group, designed for Aβ fibrils visualization.

Lim et al. [39] developed a bis(thiosemicarbazonato)copper(II) complex **1** (all numbers of coordination compounds are bold through all the manuscript) conjugated with a stilbene functional group (Figure 2). A fluorescent assay with thioflavin-T (Th-T) showed a drop in the fluorescence (485 nm) after an addition of coordination compound **1**, meaning a displacement of thioflavin. Also, examination by transmission electron microscopy (TEM) of the structural morphology of the Aβ fibrils pre-treated with coordination compound **1** showed significant changes in morphology. Epi-fluorescence microscopy of AD human brain sections with E18 antibody revealed a co-localization of the immunostained and epi-fluorescent images. Biodistribution of radiolabeled ^64^Cu-**1** in wild-type mice and *APP/PS1* transgenic mice (Tg-mice) after intravenous tail vein injection (85 MBq) showed a significantly higher brain uptake in *APP/PS1* Tg-mice compared with their wild type (Table 1).

**Figure 3:**
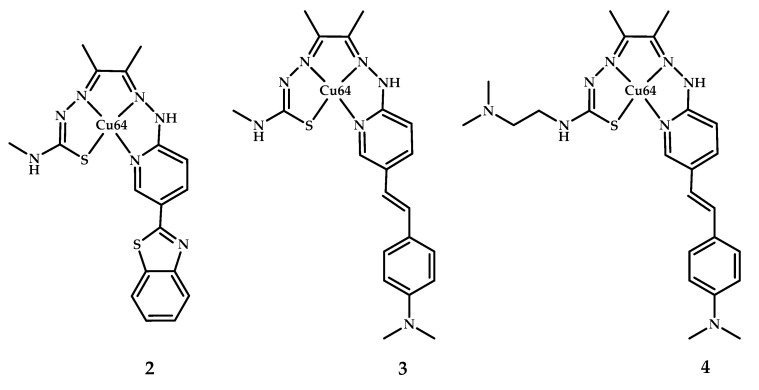
^64^Cu(II)-ATSM derivatives conjugated **2**–**4** with benzothiazole/styrylpyrydine functional group, designed for Aβ fibrils visualization.

The same Donnelly group reported a copper radiopharmaceutical Cu(II)-ATSM with an appended styrylpyridine functional group for Aβ plaque imaging [40] (Figure 3). Binding of **3** and **4** (coordination compound **2** was quite insoluble) to Aβ plaques was clearly evident, as demonstrated by epi-fluorescence microscopy. The Aβ-specific 1E8 antibody was used as a control. The biodistribution of coordination compounds **3** and **4** radiolabeled with **^64^Cu** in wild-type mice after intravenous tail injection (∼13 MBq) displayed good brain uptake of coordination compound **4** in 1.1%.

**Figure 4:**
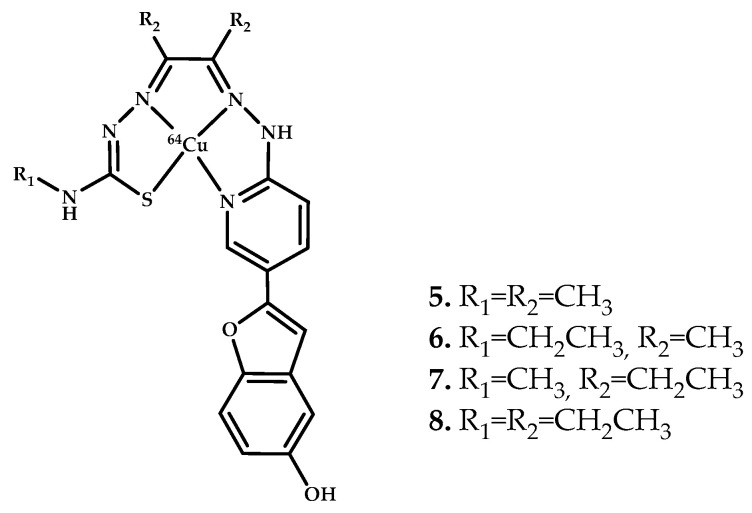
^64^Cu(II)-ATSM derivatives **5**–**8** conjugated with benzofuran functional group, designed for Aβ fibrils visualization.

In 2019 [41], the Donnely group reported a synthesis of four hybrid thiosemicarbazonato-benzofuran ligands and their copper complexes (Figure 4). Addition of either **6** or **8** to Aβ_1−42_ results in dramatic changes in the structural morphology, as identified by the TEM images. The AD human brain tissue samples treated with **8** were analyzed for elemental composition using the laser ablation inductively coupled plasma mass spectrometry (LA-ICP-MS) assay by tracking the change in the ratio ^65^Cu/^63^Cu. A sample of nonradioactive isotopically enriched **^65^Cu-8** was used to distinguish biologically present copper from the complex. Coordination compound **3** was used as a control. The benzofuran-containing complex **^65^Cu-8** appears to bind with improved differentiation compared with the styryl-pyridine-containing complex ^6**5**^**Cu-3** and potentially offers better sensitivity for amyloid. The complex preferentially binds to areas of the brain enriched with Aβ plaques, which was confirmed by immunohistochemistry with an aged-match control. The biodistribution of coordination compounds **5**–**8** radiolabeled with ^64^Cu in wild-type mice showed the best brain uptake results for coordination compound **8** (1.54% of injected dose (ID)/g at 2 min after injection, dropping to 0.77% ID/g at 30 min).

**Figure 5:**
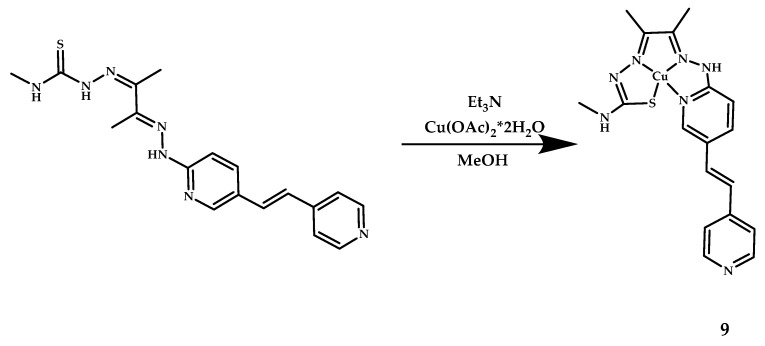
Cu(II)-ATSM derivative conjugated with piridylstilbene functional group **9**, designed for Aβ fibrils visualization.

McInne [42] incorporated a 4-vinylpyridine functional group to investigate whether the complex **9** binds to Aβ plaques with an additional pyridyl hydrogen bond acceptor at the expense of the electron-donating dimethlylamino and hydroxy groups (Figure 5). Comparing the fluorescence from the **9**-treated AD human brain tissue with (1E8)-treated brain tissue revealed good co-localization.

**Figure 6:**
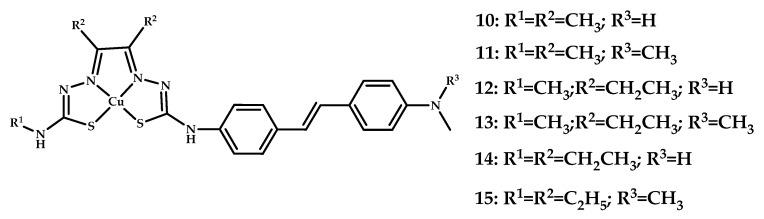
Cu(II)-ATSM derivatives conjugated with stilbene functional groups **10**–**15**, designed for Aβ fibrils visualization.

This research group recently presented several structural analogues (**10**–**15**) of coordination compound **3**, where the bis-(thiosemicarbazone) moiety is conjugated to stilbene functional groups [44] (Figure 6). All coordination compounds significantly alter the emission intensity of the ThT/Aβ conjugate. Compounds **11** and **15** were selected as lead compounds because of the ease of synthesis. The TEM of Aβ_1__−__40_ fibrils preincubated with **11** and **15** reveal a dramatic change in fibril morphology. Epi-fluorescence microscopy on human AD brain tissue proved an ability of **11** and **15** to bind amyloid-β plaques, which was also confirmed by Aβ-specific antibody (1E8) staining. Experiments with wild-type mice showed high brain uptake for both **11** and **15** at 2 min after the injection (2.2% and 1.1%, respectively), followed by rapid removal after 1 h.

Observing the various design steps of the PET binding agents developed under Donnelly’s leadership, we note that they achieved significant improvements in brain uptake (Table 1, lines 3–7).

**Figure 7:**
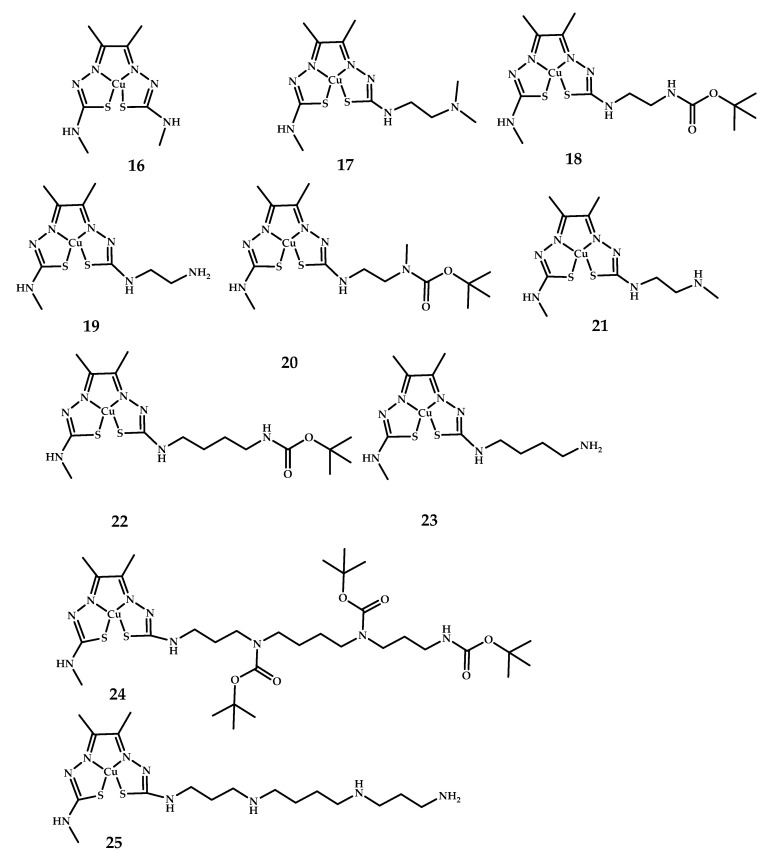
Cu(II)-ATSM derivatives conjugated with polyamines **16**–**25**, designed for Aβ fibrils visualization.

Paterson et al. [44] developed a series bis(thiosemicarbazones) **16**–**25** with amine and polyamine functional groups in order to increase the BBB permeability of the complexes (Figure 7). Intracellular uptake of the complexes was measured by inductively coupled plasma mass spectrometry (ICP-MS). Intracellular accumulation decreased in the order **17** > [**19** + 2H]^2+^ > [**21** + H]^+^ > [**23** + H]^+^ > [**25** + 3H]^3+^. Biodistribution studies were performed using small-animal micro-PET imaging. The complexes with a secondary amine, **21,** and a primary amine functional group, **23,** showed little to no radioactivity in the brain. The complex with a pendent secondary amine, **17**, had a relatively high level of brain uptake.

The authors designed these complexes not as PET imaging agents for amyloids, but as hypoxia-sensitive agents capable of accumulating in malignant tumors. But the impressive results of brain penetration shown by complex **17** (injected activity/per gramm IA/g at 23 h after injection was 2.43%) again convince us of the promising potential of copper-containing preparations as diagnostic agents for imaging brain pathologies. Ex vivo biodistribution analysis of **17**-preinjected BALB/C mice bearing EMT6 tumors showed a 4.17% ± 1.03% injected activity per gram of tissue at 40 min post-injection, and 4.41% ± 0.23% injected activity per gram of tissue in the brain.

Therefore, Cu-ATSM-based agents are interesting both as redox-active agents sensitive to hypoxia, capable of accumulation in solid tumors, and as highly penetrating agents for therapy and diagnostics of brain pathologies.

Conjugates containing Aβ-binding and metal-chelating moieties were found to modulate the aggregation of Aβ_42_ species [49,50]. Therefore, ^64^Cu coordination compounds based on them are expected to bind Aβ effectively. 

**Figure 8:**
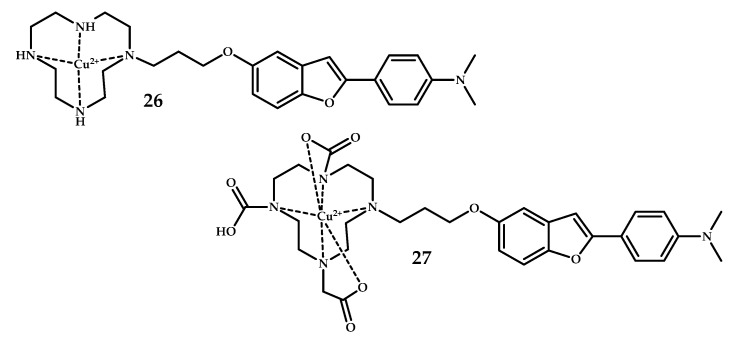
Benzofuran moiety, conjugated with metal-chelating cyclen **26** or 1,4,7,10-tetraazacyclododecane-1,4,7,10-tetraacetic acid (DOTA) **27**, designed for Aβ fibrils visualization.

Watanabe et al. designed and synthesized two novel ^64^Cu-labeled benzofuran derivatives **26** and **27** with cyclen (1,4,7,10-tetraazacyclododecane) or DOTA (1,4,7,10- tetraazacyclododecane-1,4,7,10-tetraacetic acid) as chelators [45] (Figure 8).

An in vitro binding assay with ([125I]6-iodo-2-(40-dimethylamino)-phenyl-imidazo [1,2-a]pyridine) [^125^I] IMPY as the competitive ligand showed dose-dependent inhibition with K_i_ 33.7 ± 14.6, 243.5 ± 88.2. Fluorescent staining using Tg2576 mice brain sections proved the amyloid-binding ability of **26** to a greater extent than **27**. Unfortunately, biodistribution studies revealed quite low brain uptake equal to 0.33% and 0.36%, respectively.

**Figure 9:**
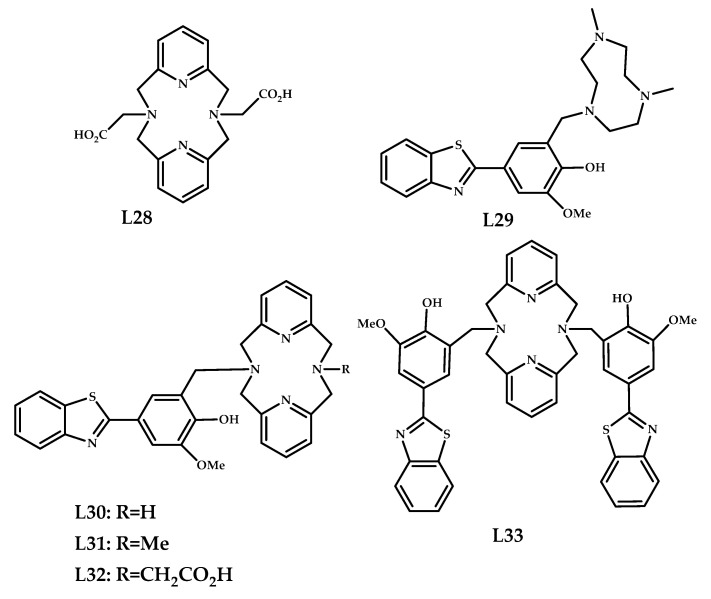
Benzothiazole moieties, conjugated with metal-chelating 1,4,7-triazacyclononane and 2,11-diaza[3.3]-(2,6)pyridinophane **L29**–**L33**, designed for Aβ fibrils binding, and model ligand **L28** without benzotiazole moiety.

Sharma et al. designed a series of copper-coordination compounds based on an Aβ-binding 2-phenylbenzothiazole moiety, conjugated with metal-chelating macrocyclic 1,4,7-triazacyclononane (tacn) and 2,11-diaza [3.3]-(2,6)pyridinophane (N_4_H_2_) **29**–**33** [46,47] (Figure 9). The ThT fluorescence competition assay suggests a good affinity **L29**–**L33** for Aβ_40_ fibrils. Fluorescence microscopy studies on *Tg2576 APP* Tg-mice brain sections, with amyloid-binding Congo Red as a control, showed a specific binding for organic ligands **L29**–**L33**. The ThT competition assays with copper complexes **29**–**33** also revealed a strong Aβ binding affinity for **32**. A specific binding of the ^64^Cu-labeled **L29**–**L33** to Aβ plagues was proven using ex vivo autoradiography studies on brain sections of Tg2576 mice and wild-type mice as a control in the absence and presence of a known Aβ-specific blocking agent (B1). Coordination compounds **29**–**33** showed a significant Aβ binding: the autoradiography intensity markedly decreased in the presence of B1 blocking agent. Biodistribution studies in normal CD-1 mice showed the highest brain uptake of 1.33% ± 0.27% ID/g at 2 min post-injection for **29**. The PET/CT imaging of the Tg2576 mice showed a radiotracer accumulation in the head and neck area for **29**, **31**, and **32**. Coordination compound **29** shows the highest brain uptake of 0.57% ± 0.05% ID/g in post-PET biodistribution analysis.

**Figure 10:**
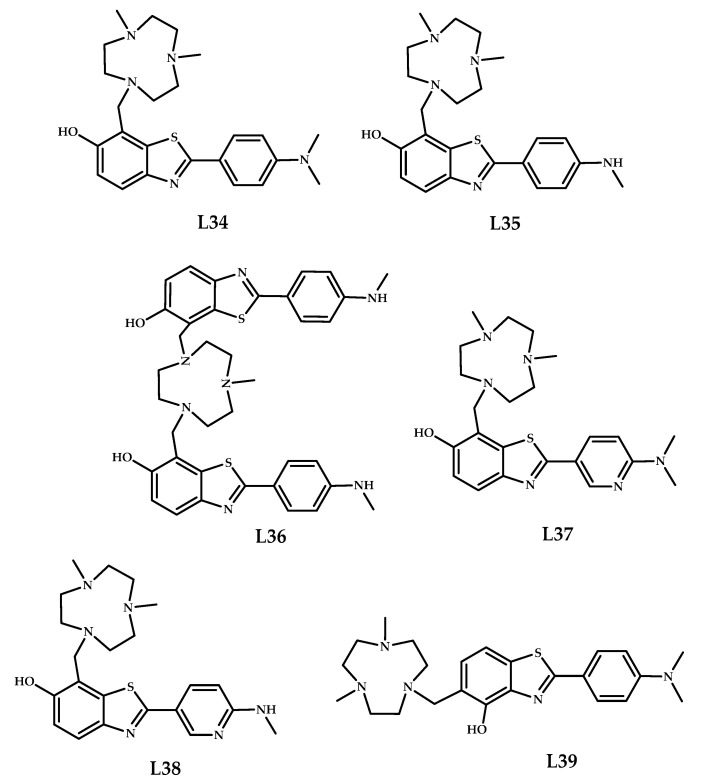
Pittsburg compound B derivatives, conjugated with metal-chelating 1,4-dimethyl-1,4,7-triazacyclononane **L34**–**L39**, designed for Aβ fibrils binding.

Huang et al. developed a series of compounds based on classical amyloid-binding moiety Pittsburg compound B and used fragments 1,4-dimethyl-1,4,7-triazacyclononane (tacn) as the metal-chelating group [48] (Figure 10). The ThT fluorescence competition assays showed nanomolar affinities for the Aβ_1–40_ for organic ligands L**34** and L**35**. Staining with 5xFAD mice brain sections showed significant Aβ-binding affinity of the organic ligands L**34**–**36** and L**39**. The Cu^2+^ complexes **35**, **36**, and **39** also showed significant Aβ binding. The MTT (3-(4,5-dimethylthiazol-2-yl)-2,5-diphenyl-2H-tetrazolium bromide) cell viability assays on mice neuroblastoma (N2a) cells showed that coordination compounds **35**, **37**, and **38** exhibit no appreciable cell toxicity. Unfortunately, determination of the octanol/phosphate-buffered saline (PBS) partition coefficient values revealed that ^64^Cu-labeled complexes **37** and **38** exhibit log D_oct_ values of 0.6, suggesting that 2-pyridyl-benzothiazole derivatives may be too hydrophilic to cross the BBB.

Ex vivo autoradiography studies using brain sections of 5xFAD Tg-mice confirmed an amyloid-binding specificity of radiolabeled coordination compounds **35**, **36**, and **39**, but **^64^**Cu-labeled **34** also exhibits nonspecific binding. The MW of **36** was found to be too large for efficient brain uptake. Biodistribution studies in normal CD-1 mice proved **39** to cross the BBB, while **35** showed low brain uptake.

## 3. Gd^3+^ and Ga^3+^ Coordination Compounds for Aβ Visualization

Another promising emerging radionuclide for PET is ^68^Ga. Positron-emitting ^68^Ga can be obtained from a ^68^Ge/^68^Ga generator, which would facilitate cyclotron-independent distribution of PET. The parent nuclide ^68^Ge has a half-life of 271 days, and the generators can provide sufficient quantities of ^68^Ga for up to one year, resulting in a relatively inexpensive and reliable source of a positron-emitting radionuclide [51]. Ga^3+^ is a hard acid metal that can make strong bonds with hard base ligands such as carboxylic acids, amino nitrogen hydroxamates, and phenolates [52], which leads to the tendency to use rigid oxygen-containing chelating structures in ^68^Ga-based drug candidates, such as 1,4,7,10-tetraazacyclododecane-1,4,7,10-tetraacetic acid DOTA.

**Figure 11:**
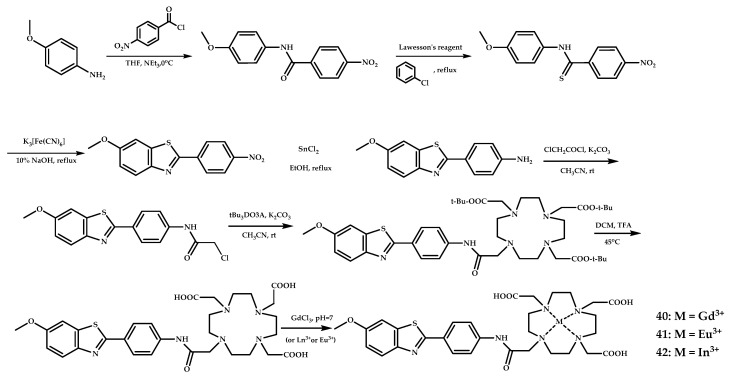
Synthesis of Aβ-specific DO3A-benzothiazole ligand **L40** and coordination compounds **40** (Gd^3+^), **41** (Eu^3+^), and **42** (In^3+^) based on it, designed for MRI and SPECT Aβ fibrils visualization.

MRI is an imaging technique based on the physical phenomenon of nuclear magnetic resonance. Various structural and functional changes including atrophy, vascular dysfunction, or changes in the volume of the hippocampus can be quantified using anatomical MRI [53]. Gadolinium(III) is the constituent of most MRI contrast agents due to a large magnetic moment (spin only effective magnetic moment μ_eff_ ¼ 7.94 BM, from seven half-filled f-orbitals) and a long electron-spin relaxation time (108 to 109 s, from the symmetric S electronic state) [54]. Table 2 summarizes the coordination compounds for magnetic resonance imaging (MRI) and single-photon emission computed tomography (SPECT) diagnostics of Alzheimer’s disease, based on amyloid-affinity ligands conjugated with various metal chelating moieties:

Martins et al. have designed an amyloid-targeted ligand that can efficiently complex different metal ions for various imaging modalities, including Gd^3+^ for MRI and ^111^In^3+^ for SPECT imaging by a conjugation of a cyclen-based macrocycle DO3A (1,4,7,10-tetraazacyclododecane-1,4,7-triacetic acid) with a benzothiazole moiety [55]. Ligand **L40**-based complexes of Gd^3+^, Eu^3+^, and ^111^In^3+^ were obtained (Figure 11).

Upon binding of **40** to Aβ plaques, higher relaxivity in nuclear magnetic relaxation dispersion (NMRD) profiles was observed due to the complex becoming immobilized during plaque binding. A binding affinity of **40** to Aβ_1−40_ was evaluated by surface plasmon resonance measurements and yielded K_d_ = (180 ± 10) μM, and similar K_d_ values were also expected for the Eu^3+^ and In^3+^ analogues **41** and **42**. The binding affinity of **40** to HSA was assessed by proton relaxation enhancement measurements and yielded K_d_ = 110 ± 20 μM. A specific binding of **41** to Aβ deposits was proved on postmortem human brain tissue of AD patients using fluorescence staining with PiB and thioflavin-S as controls. Unfortunately, the log P oct/water −0.15 value for **40** and also the high MW = 842 shows that the complex is not optimized to cross the BBB. In vivo biodistribution experiments with the radiolabeled ^111^In-analogue **42** in adult male Swiss mice showed that cortex and cerebellum penetration ID/g at 2 min was 0.36% and 0.5%, respectively.

**Figure 12:**
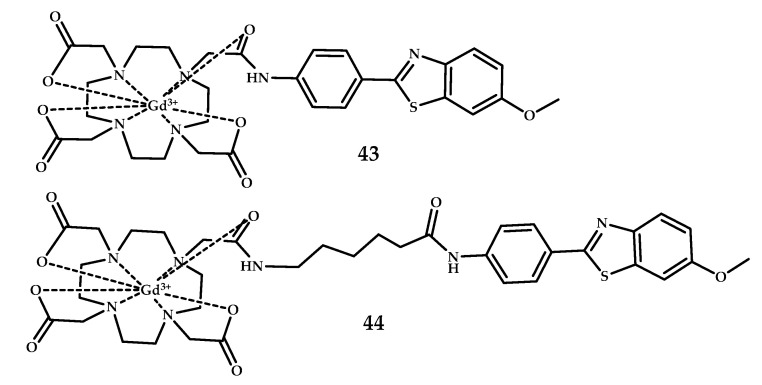
DO3A-PiB-based Gd^3+^ coordination compounds **43** and **44**, designed for MRI visualization of Aβ plagues.

Martins et al. subsequently presented two novel DO3A monoamide derivative ligands conjugated to the PiB moiety, **43** and **44**, via linkers differing in length and chemical structure to improve the log P-value and to enhance BBB penetration of the complexes [56] (Figure 12).

The amphiphilic compounds **43** and **44** were found to form micelles in solution. Analysis of the rotational dynamics for micelles formed using the Lipari-Szabo approach indicated highly flexible large aggregates. The coordination compounds **43** and **44** were unable to cross the BBB, and the amount detected was found to be insufficient for MRI detection.

**Figure 13:**
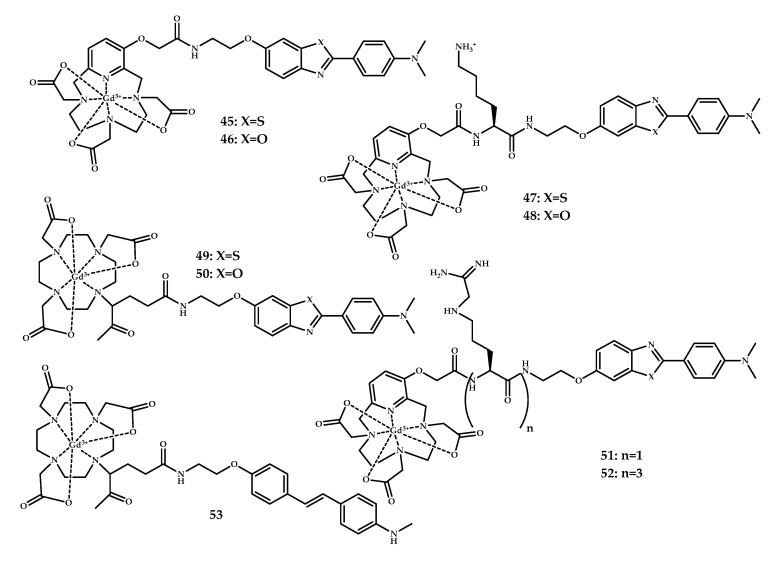

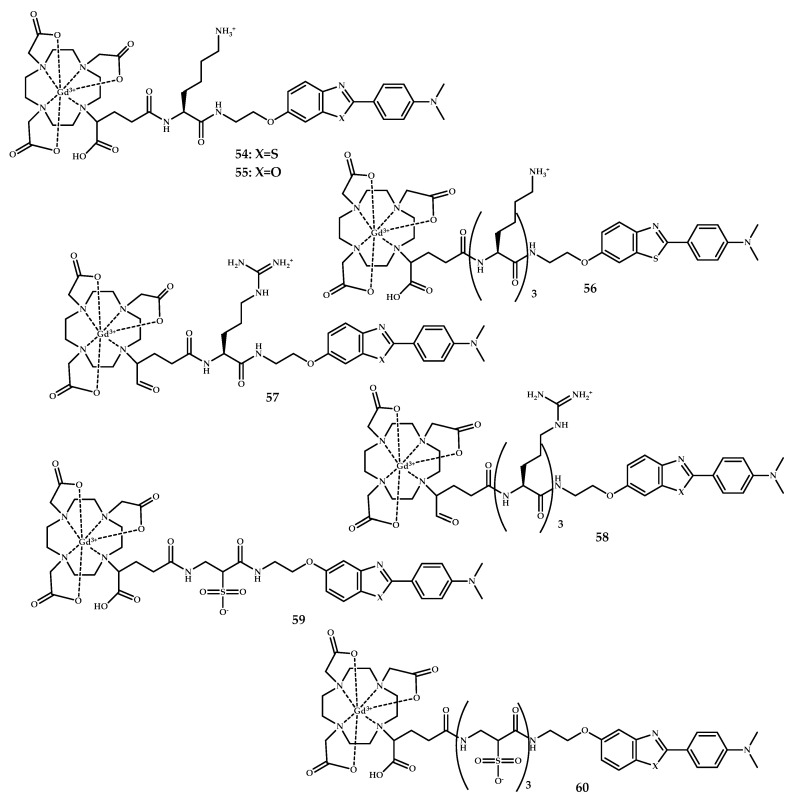
PCTA/DOTA-benzothiazole/benzoxazole/stilbene-based Gd^3+^ coordination compounds **45**–**60** designed for MRI visualization of Aβ plagues.

Bort et al. reported amyloid-targeted hydroxybenzothiazole, hydroxybenzoxazole, and hydroxy-trans-stilbene moieties conjugated via neutral and positive-charged linkers with PCTA (3,6,9,15-tetraaza bicyclo[9.3.1]-pentadeca1(15),11,13-triene-3,6,9-triacetic acid) and DOTA (1,4,7,10-tetraazacyclododecane-1,4,7,10-tetraacetic acid) as metal-chelates, and Gd(III) complexes **45**–**60** based on them [57] (Figure 13).

The affinity of the coordination compounds **45**–**60** for amyloid aggregates was determined in vitro using [^125^I]IMPY ([^125^I]6-iodo-2-(40-dimethylamino)-phenyl-imidazo [1,2-a]pyridine)-binding competition experiments on synthetic Aβ_1–42_ aggregates, with DOTA-(Lys)_3_-BTA being the most potent. To assess the BBB permeability of the coordination compounds, an in vitro model of BBB constituted of a co-culture of rat primary brain capillary endothelial cells and rat glial cells was used. Unfortunately, none of the designed complexes showed BBB penetration ability.

**Figure 14:**
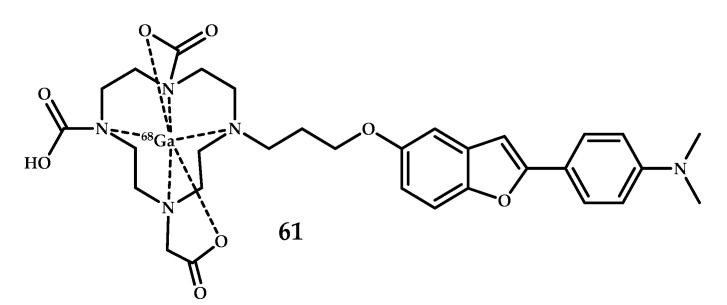
DOTA-benzofuran-based Gd^3+^ coordination compound **61** designed for MRI visualization of Aβ plagues.

Watanabe et al. designed and synthesized ^68^Ga-labeled benzofuran derivative **61** with 1,4,7,10-tetraazacyclododecane-1,4,7,10-tetraacetic acid (DOTA) as the metal-chelating agent [58] (Figure 14). A competitive Aβ_1–42_ binding experiment of **61** (with [^125^I] (IMPY) as the competitive ligand) showed a dose-dependent inhibition and values close to the clinically applied IMPY. Neuropathological fluorescent staining of Tg2576 mice brain sections treated with coordination compound **61** with Thioflavin S as a control proved a specific binding of the coordination compound to Aβ plaques. A biodistribution experiment in normal mice showed brain uptake of the coordination compound **61** (0.45% ID/g), which is too low for the compound to serve as an MRI agent.

**Figure 15:**
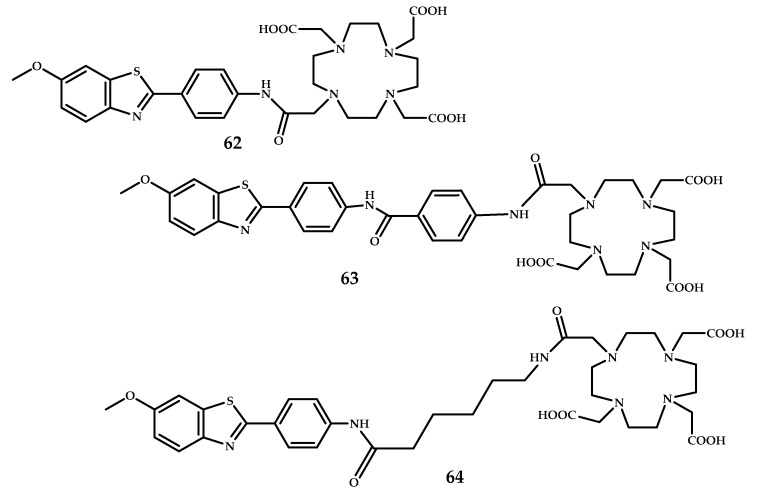
DOTA-Pib-based ligands **L62**–**L64**.

Cressier et al. reported ^68^Ga-labeled complexes conjugated to Pittsburgh Compound B, 2-(4′-[^11^C]methylaminophenyl)-6-hydroxybenzothiazole (PIB) and DOTA via aromatic or alkyl pacers **L62**–**L64** [59] (Figure 15). The BBB permeability of the complexes was insufficient, as shown by µPET. Moreover, the evaluation of the complexes **62**–**64** through an autoradiographic approach with human brain tissues failed to detect amyloid deposits.

**Figure 16:**
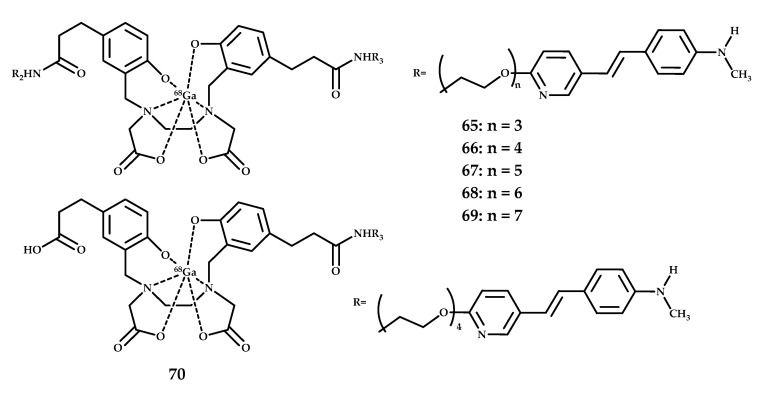
HBED-CC-styrilpiridine coordination compounds **65**–**70**, designed for PET imaging of Aβ plaques.

Zha et al. reported ^68^Ga-labeled styrylpyridine derivatives **65**–**70** with high MW based on an N,N’-bis[2-hydroxy-5-(carboxyethyl)benzyl]ethylenediamine-N,N’-diacetic acid (HBED-CC) core for Ga^3+^ complexation derivatized with styrylpyridinyl groups [60] (Figure 16). An in vitro competitive binding assay was conducted to measure the inhibition of [^125^I]IMPY Aβ binding by coordination compounds **65**–**70**. The monovalent conjugate **69** showed a low binding affinity. The in vitro autoradiography on AD brain sections showed a high binding affinity of **65**–**70** to Aβ plaques, but in vivo biodistribution studies in CD-1 mice showed low brain penetration. This may allow a selective labeling of Aβ plaques deposited on the walls of cerebral blood vessels, which could be a useful tool for diagnosing cerebral amyloid angiopathy (CAA), but not in the Aβ plaques in the parenchymal brain tissues.

Curcumin (C21), (1E,6E)-1,7-bis(4-hydroxy-3-methoxyphenyl)-1,6-heptadiene-3,5-dione, is a promising organic motif for designing biologically active coordination compounds. Curcumin demonstrated high antiproliferative activity in vitro and in vivo [67] and is also known to accumulate in tumor cells, presumably due to the ability to bind the vitamin-D receptor [68].

Curcumin and its derivatives are widely studied as agents for diagnosis, prevention, and treatment of AD [69,70], and also proved to be an amyloid-specific dye [71,72]. It binds to soluble Aβ plagues [73] and is reported to have sufficient brain permeability and favorable amyloid-binding in *APPsw* Tg-mice [74]. Curcumin is currently regarded as a specific organic core for AD therapy and diagnostic drug development. Several curcumin-based fluorescent probes for Aβ imaging have been designed [75]. A number of research works are devoted to a curcumin-based metal-containing agent for MRI, SPECT, and PET diagnostics [76].

**Figure 17:**
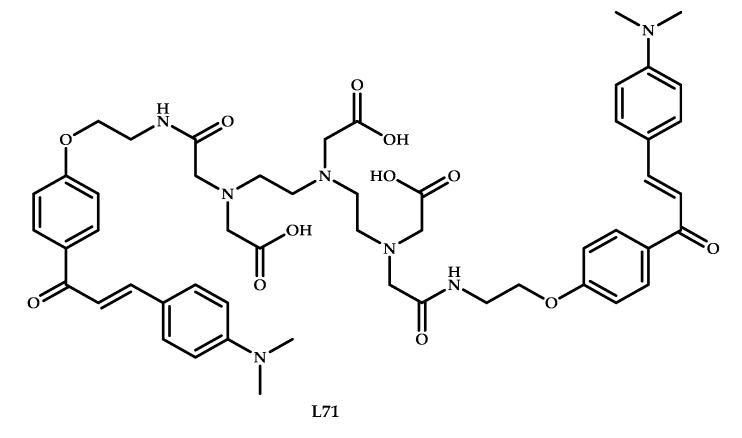
Chalchone-based ligand **L71,** designed for Aβ plaques binding.

The affinity of curcumin for amyloid plaques has raised interest in chalcone derivatives as organic core for the development of Aβ-affinity diagnostic agents. In 2007, Ono et al. reported chalcone-based probes for in vivo imaging of Aβ plaques in Alzheimer’s brains [77]. Chauhan et al. reported a bis-chalcone Ga^3+^-based coordination compound **71** [61] (Figure 17). The stability of coordination compound **69** in HSA was proven using ITLC-SG. Also, the high Aβ-binding affinity of **69** to HAS was proven in a protein-binding assay. Aβ-binding studies on aggregated Aβ_42_ were performed, and Scatchard plots suggest one-site binding with a K_d_ of 3.46 ± 0.41 nM.

Blood kinetics studies of coordination compound **71** in normal rabbits showed a fast clearance during the initial time period of 30 min. Biodistribution studies showed a high uptake level of 1.24% ± 0.31% with rapid excretion within an hour. Also, PET images in a normal adult male BALB/C mice during 2–30 m intravenous post-injection exhibited a significant activity in the brain at 2 min post-injection and rapid washout from the healthy brain. Thus, coordination compound **71** showed no specific binding or prolonged retention in the healthy brain, due to the absence of Aβ plagues.

**Figure 18:**
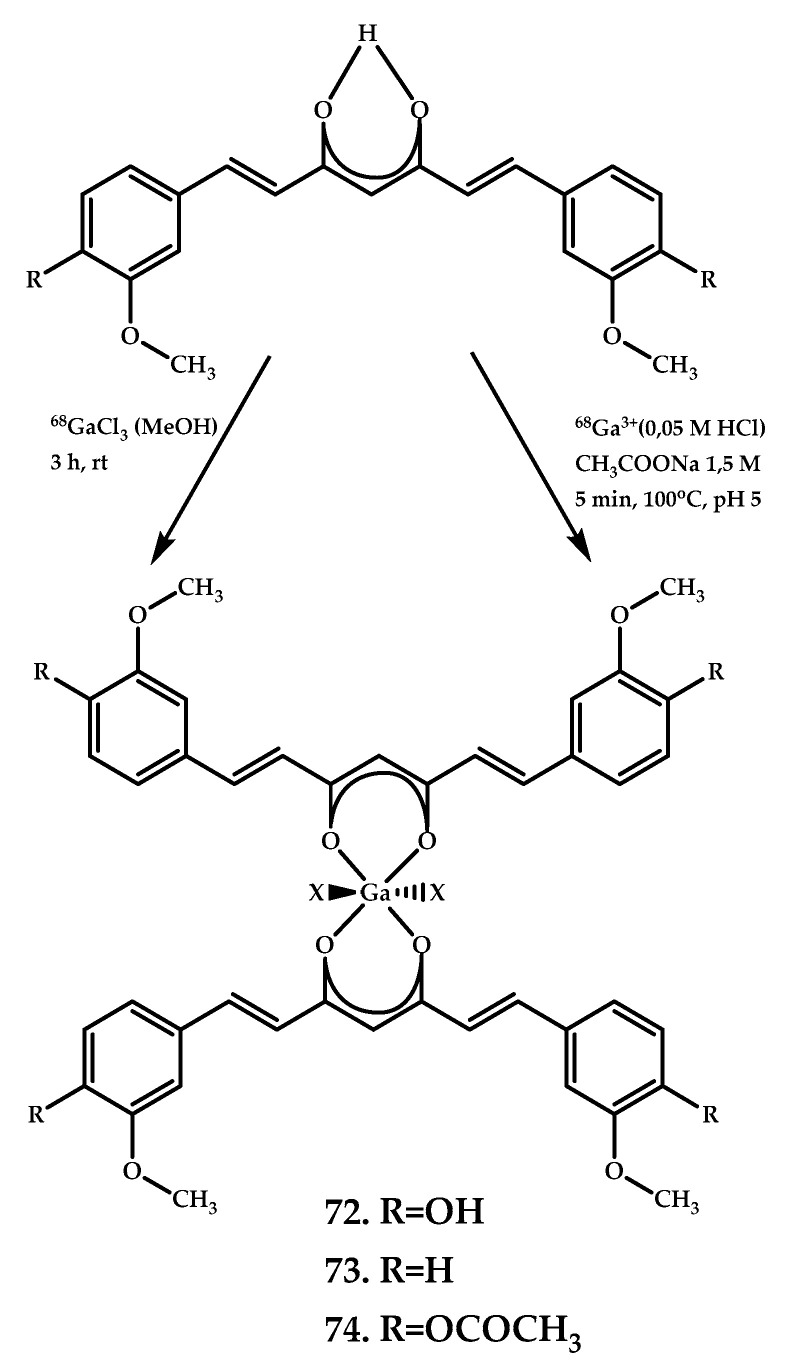
Curcumin-based Ga^3+^ coordination compounds **72**–**74**, designed for PET imaging of Aβ plaques.

Asti et al. reported ^68^Ga-labeled complexes based on curcumin, diacetyl-curcumin (DAC), and bis(dehydroxy)curcumin (bDHC) **72**–**74** [62] (Figure 18). The affinity of nat/^68^Ga-Curcuminoid complexes **72**–**74** for Aβ_1−40_ amyloid synthetic fibrils was evaluated by measuring the radioactivity of synthetic Aβ fibrils preincubated with complexes **72**–**74** and also using fluorescence microscopy with untreated fibrils as a negative control. A fluorescence microscopy study of drug-preincubated A-549 tumor cells confirmed an internalization of Ga^3+^-curcuminoid complexes in lung cancer cells.

Continuing the study, Rubagotti et al. reported [63] an in vitro and in vivo investigation of the biological properties of coordination compounds **72**–**74**. The in vivo brain uptake was assessed using a Tg2576 mice model. Although Aβ plagues were clearly visualized after brain section staining with coordination compounds, no brain uptake in vivo was observed. These results indicate a high Aβ-affinity of gallium complexes **72**–**74** along with an inability of the coordination compounds to cross the BBB in vivo.

**Figure 19:**
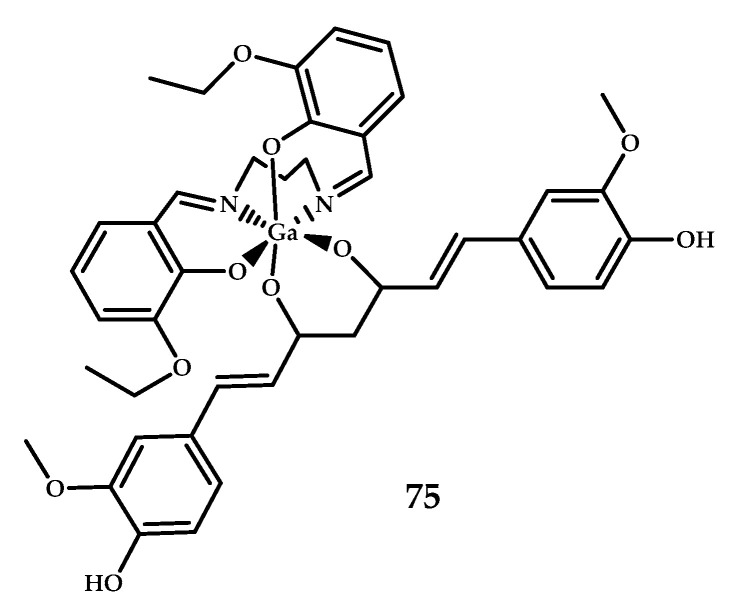
Curcumin-based Ga^3+^ coordination compound **75** with a Schiff-based metal-chelating moiety, designed for PET imaging of Aβ plaques.

Lange et al. reported [64] a six-coordinate Ga^3+^ complex **75** based on an N_2_O_2_ Schiff-base ligand and β-diketone curcumin, which is known to bind to Aβ plagues because of the structural similarity to Congo Red [78] (Figure 19). The ability of **75** to bind to Aβ plaques was assessed using epi-fluorescence microscopy (λex = 359 nm, λem = 461 nm) on AD and age-matched human brain samples with an 1E8-antibody as control. The obtained results allow suggesting some degree of specificity of **73** for Aβ plaques.

**Figure 20:**
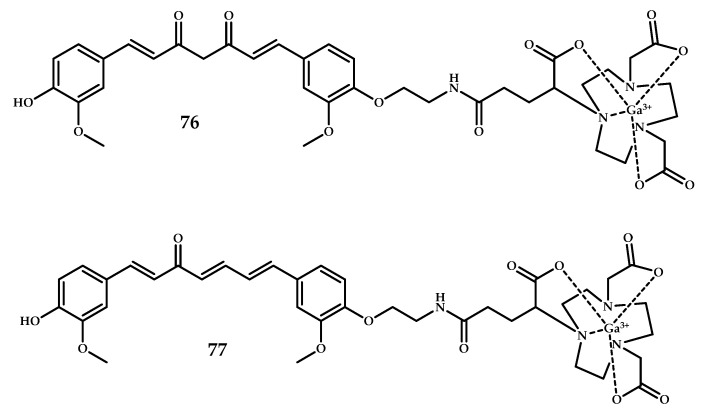
Curcumin-based Ga^3+^ coordination compounds **76** and **77** with NODAGA and AAZTA metal-chelating moieties, designed for PET imaging of Aβ plaques.

Orteca et al. recently reported curcumin scaffolds conjugated with 1,4,7-triazacyclononane,1-glutaric acid-4,7-acetic acid (NODAGA) and 1,4-bis(carboxymethyl)-6-[bis(carboxymethyl)]amino-6-methylperhydro-1,4-diazepine (AAZTA) as metal chelators **L76** and **L77** [65] (Figure 20).

**Figure 21:**
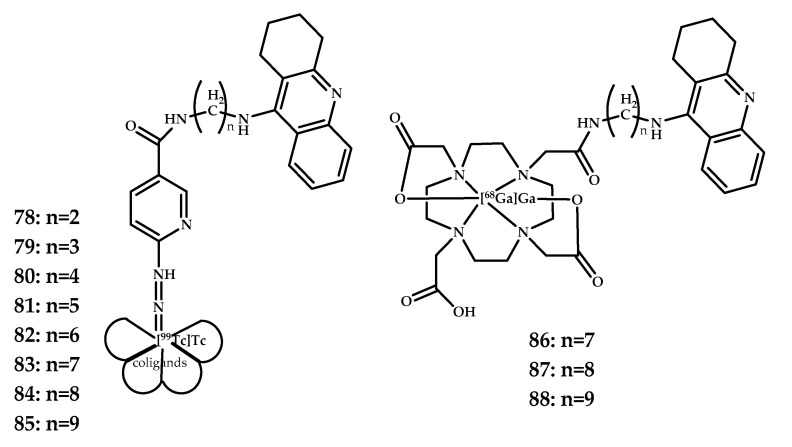
Tacrine-based ^99m^Tc^3+^ coordination compounds **78**–**85** and Ga^3+^ coordination compounds **86**–**88** with Hynic and DOTA metal-chelating moieties, designed for PET imaging of Aβ plaques.

Gniazdowska et al. designed a series of tacrine analogues, acetylcholinesterase (AChE) and butyrylcholinesterase (BuChE) inhibitor [79], the enzymes responsible for the degeneration of the neurotransmitter acetylcholine and labeled with diagnostic radionuclides technetium-99m using bifunctional ligand Hynic [80] **78**–**85**, and gallium-68, using macrocyclic ligand DOTA **84**–**86** [80] (Figure 21). The Log D values for the coordination compounds are presented in Table 3. Coordination compounds **82** and **86** with the highest Log D values were selected as lead compounds.

An ability of coordination compounds **82** and **86** to inhibit acetylcholinesterase (AChE) and butyrylcholinesterase (BuChE) was estimated using Ellman’s colorimetric assay. The half maximal inhibitory concentration IC_50_ values for the tested derivatives are presented in Table 4. Tacrine was used as the reference inhibitor.

An in vivo pharmacodynamic study of coordination compound **86** allowed only a qualitative view because the brain penetration was low, 0.21%. The pharmacodynamic study of coordination compound **82** was incomplete due to the low activity of the compound, and the result was therefore omitted. But the ex vivo radioactivity measurement showed that both complexes can penetrate the BBB.

## 4. ^99m^Tc^3+^-Based Coordination Compounds for SPECT Visualization of Aβ

**Figure 22:**
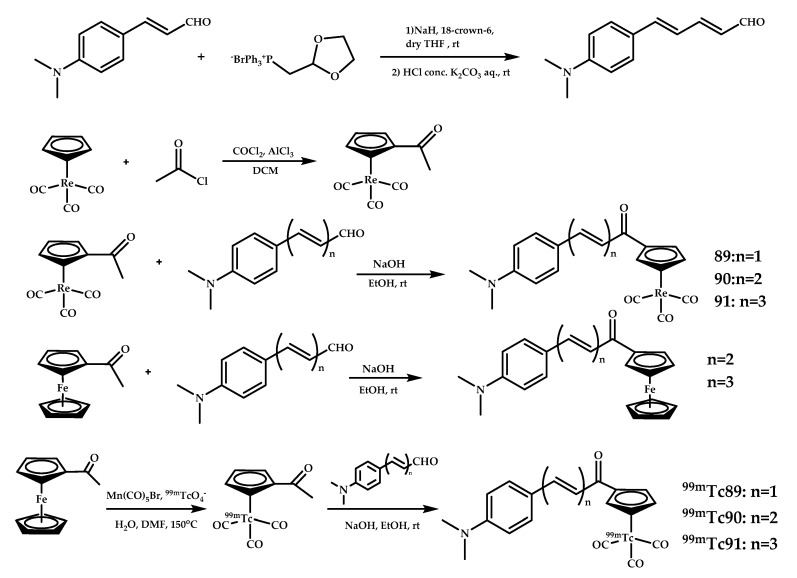
[^99m^Tc] coordination compounds **[^99m^Tc]89**–**91** based on chalchone-mimic scaffolds and their Re analogues **89–91**.

To overcome the limitations of PET imaging in terms of cost and broad accessibility, SPECT was proposed as alternative diagnostic tool [81]. Technetium-99m (^99m^Tc) is a desirable radioisotope for the preparation of SPECT radiopharmaceuticals because it has a rich chemistry, unique nuclear properties (T_1/2_ = 6 h, E = 140 keV), and an easy cost-effective availability. ^99m^Tc can be readily prepared by a ^99^Mo/^99m^Tc generator [82]. The development of a ^99m^Tc-radiotracer for imaging Aβ plaques with SPECT is strongly expected to provide a low cost, broadly accessible diagnostic tool for AD. Table 5 summarizes the coordination compounds for single-photon emission computed tomography (SPECT) diagnostics of Alzheimer’s disease:

Liu et al. designed and synthesized novel chalcone-mimic Re/^99m^Tc **Re-89–91/[^99m^Tc]87–91** complexes [83] (Figure 22). Ferrocene complexes were synthesized as precursors for ^99m^Tc coordination compounds. Complexes **Re-90** and **Re-91** demonstrated a high affinity to Aβ plaques in brain tissue sections from AD patients and Tg-mice (*APPswe/PSEN1*), while demonstrating no apparent labeling in both normal mice C57BL6 and normal adult brain sections. The K_i_ value ranges established using an Aβ_1__−__42_ binding assay ranged from 899 to 108 nM. As an extension of the conjugated π system, complex **Re-91** demonstrated the highest affinity. The in vitro autoradiography of **[^99m^Tc]89–91** on Tg-mice brains confirmed the Aβ affinity of **[^99m^Tc]91** (Ki = 108 nM). In the biodistribution studies, **[^99m^Tc]89** and **[^99m^Tc]90** showed excellent initial uptakes and fast clearance (respectively 4.10% and 2.30%) in the brain, while **[^99m^Tc]91** showed moderate brain uptake (1.11%).

A biodistribution in permeability-glycoprotein blocked by cyclosporin A (an immunosuppressant drug) revealed an increase of BBB-penetrating abilities of the coordination compounds **[^99m^Tc]89–91**. This result may reveal **[^99m^Tc]89–91** to be substrates for the rodent PgP transporter.

**Figure 23:**
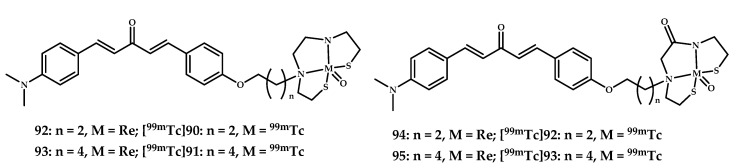
Coordination compounds **Re/^99m^Tc 92**–**95** based on dibenzylideneacetone scaffolds with BAT (**92, 93/[^99m^Tc]92, [^99m^Tc]93**) and MAMA (**94, 95/[^99m^Tc]94, [^99m^Tc]95**) designed for SPECT imaging of Aβ plaques.

Yang et al. reported four ^99m^Tc-labeled dibenzylideneacetone derivatives **[^99m^Tc]92**–**95** and corresponding rhenium complexes **92**–**95** [84] (Figure 23).

The binding affinities of rhenium complexes **92**–**95** for Aβ_1–42_ aggregates were evaluated by competition binding assay using [^125^I]IMPY. Coordination compounds **92** and **93** with the BAT chelating moiety showed better Aβ_1–42_ affinity (K_i_ = 24.7 and 13.6 nM) compared with coordination compounds **94** and **95** with the MAMA chelating moiety (K_i_ = 120.9 and 59.1 nM). Increasing the length of the spacer was found to promote Aβ_1–42_ binding. All four rhenium complexes, **92**–**95**, displayed excellent labeling of Aβ plaques in in vitro fluorescent staining on sections of brain tissue from a Tg-mice (C57BL6, *APPswe/PSEN1*) and age-matched control mice. Biodistribution experiments of ^99m^Tc-labeled coordination compounds **[^99m^Tc]92**–**95** in normal ICR mice showed the highest initial uptake at 2 min post-injection (respectively 0.49%, 0.47%, 0.48%, and 0.31% ID/g), followed by rapid washout from the brain.

**Figure 24:**
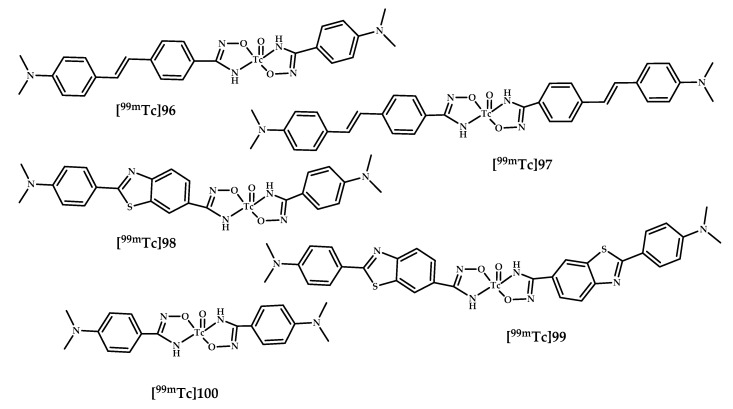
^99m^Tc–HAM complexes based on stilbene and benzothiazole moieties **[^99m^Tc]96**–**99**, designed for SPECT imaging of Aβ plaques, and model coordination compound **100**.

Iikuni et al. designed five novel ^99m^Tc-Ham complexes **[^99m^Tc]96**–**99** with a bivalent amyloid ligand based on stilbene/benzothiazole moieties and HAM as chelating agent [85] (Figure 24).

Coordination compounds **[^99m^Tc]96**–**99** displayed moderate affinity for amyloid aggregates (respectively 22.2%, 42.6%, 4.6%, 38.7%), while model compound **[^99m^Tc]100**, which does not include any amyloid ligands, showed no affinity. In vitro autoradiography of Tg2576 mice brain section assay proved an ability of **[^99m^Tc]96**, **[^99m^Tc]97**, and **[^99m^Tc]99** to bind Aβ plaques. A biodistribution experiment of **[^99m^Tc]97** with the highest binding affinity in the inhibition assay in normal mice showed very low brain uptake (0.28% ID/g).

Further, the authors of Reference [86] applied coordination compounds **[^99m^Tc]96**–**99** to CAA-specific imaging probes and evaluated their utility for CAA-specific imaging. An in vitro inhibition assay using Aβ_1–40_ aggregates deposited mainly in CAA showed a high binding affinity of coordination compounds **[^99m^Tc]96**–**99**. In vitro autoradiography of human CAA brain sections and ex vivo autoradiography of Tg2576 mice displayed excellent labeling of Aβ depositions in human CAA brain sections and high affinity and selectivity to CAA in Tg-mice of coordination compounds **[^99m^Tc]97** and **[^99m^Tc]99**.

**Figure 25:**
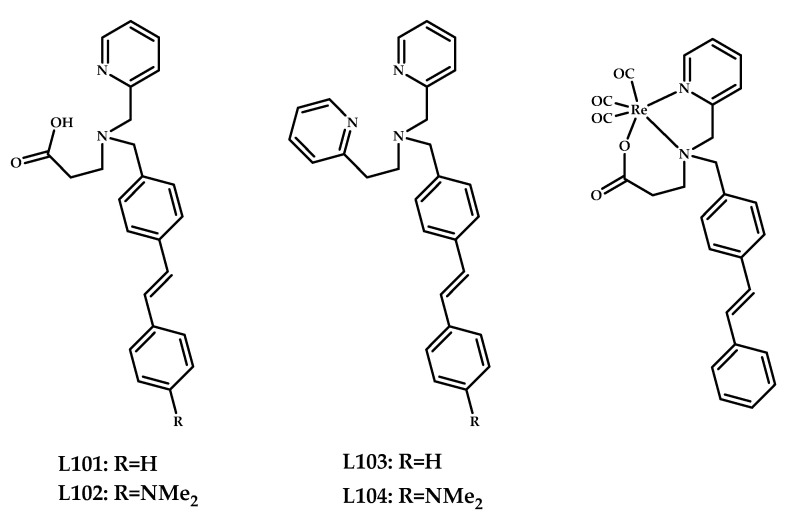
Tridentate ligands **L101**–**L104** conjugated with a stilbene Aβ-binding moiety designed for Aβ plaques binding, and the proposed structure of coordination compound **101**.

Hayne et al. reported [87] tridentate ligands **L101**–**L104** designed to bind to the [M(CO)_3_]^+^ core (M = Tc/Re) conjugated with a stilbene Aβ-binding moiety (Figure 25). The complexes **101** and **103** showed little to no plaque binding in brain tissue from AD-positive subjects. Epi-fluorescence microscopy of tissue sections of the frontal cortex of an AD-affected brain treated with **102** and **104** bearing an electron-donating dimethylamino functional group revealed good correlation of the complexes to Aβ plaques, and the E18 antibody was used as a control.

The biodistribution of the radiolabeled coordination compound **[^99m^Tc]103** was investigated in both wild-type and *APP/PS1* Tg-mice. Low brain uptake (~0.25%) was registered in both cases, and no statistically significant difference between wild-type and Tg-mice was observed.

**Figure 26:**
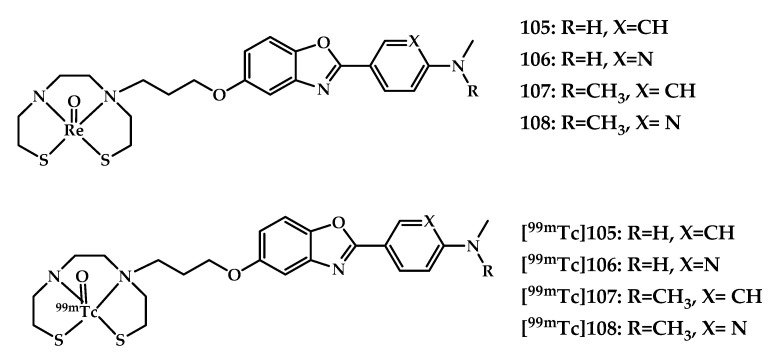
Re^3+^ (**105**–**108**) ^99m^Tc^3+^ ([**^99m^Tc]105–108**) complexes based on arylbenzoxazole with a BAT metal-chelating moiety, designed for SPECT imaging of Aβ plaques.

Wang et al. reported four neutral Re/^99m^Tc-labeled coordination compounds **105–108**/**[^99m^Tc]105–108** based on arylbenzoxazole moieties conjugated with bis(aminoethanethiol) (BAT) as a chelating moiety [88] (Figure 26).

In vitro fluorescent staining with rhenium complexes **105**–**108** with Aβ plaques, neuropathological staining with the brain sections of a Tg-mice and an AD patient showed specific Aβ-binding of the complexes. An in vitro competition binding assay was performed using [^125^I] IMPY as the competing radioligand. A moderate Aβ-binding affinity of **105** and **106** (K_i_ = 128.21 and 393.18 nM) and a high affinity of complexes **107** and **108** (K_i_ = 15.86 and 37.19 nM) with N,N-dimethyl amino group was estimated. ^99m^Tc-labeled complexes were prepared by a ligand exchange reaction from the intermediate ^99m^Tc-glucoheptonate. In vitro autoradiography in Tg-mice brain tissue showed labeling of cortex, hippocampus, and cerebellum regions by [**^99m^Tc]107**. Biodistribution studies of coordination compounds displayed higher initial brain uptake of N,N-dimethylated derivatives and brain_2min_/brain_60min_ ratio than the N-monomethylated analogs ([**^99m^Tc]105** vs [**^99m^Tc]107** and [**^99m^Tc]106** vs [**^99m^Tc]108**).

**Figure 27:**
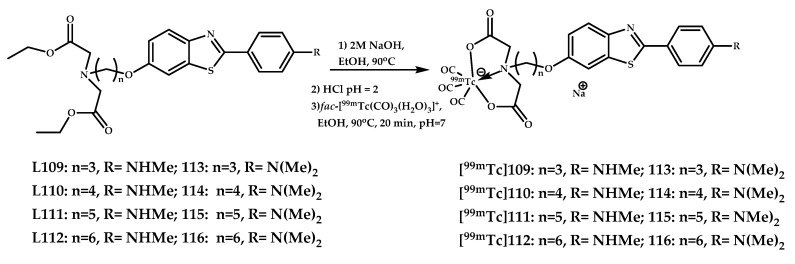
Negatively charged imaging probes [**^99m^Tc]109–116** designed for the selective detection of vascular Aβ deposition, and their Re^3+^ analogues **109**–**116**.

Jia et al. reported a design and biological evaluation of a series of negatively charged imaging probes with limited BBB penetration for the selective detection of vascular Aβ deposition [89]. Eight ^99m^Tc(CO)_3_-labeled benzothiazole derivatives [**^99m^Tc]109–116** and their Re(III) analogues **109**–**116** were designed as potential SPECT imaging probes for cerebrovascular Aβ deposition (Figure 27). Rhenium surrogates **109**–**116** displayed high affinities to Aβ aggregates with K_i_ values ranging from 42 to 106 nM, rhenium complex **116** with the longest carbon linker length (*n* = 6) displayed the highest affinity to Aβ_1−42_ aggregates (K_i_ = 42.2 nM). Complex **115** also demonstrated unambiguous and specific labeling of Aβ plaques in brain sections from Tg-mice. ^99m^Tc-labeled coordination compounds [**^99m^Tc]109–116** were obtained by ligand exchange reactions with fac–[^99m^Tc(CO)_3_(H_2_O)_3_]^+^.

Autoradiography studies in AD human brain tissue proved the ability of coordination compound [**^99m^Tc]116** to bind Aβ deposits in blood vessels but not in cerebral parenchyma on brain sections of an AD patient, while [^125^I]IMP labeled both. Ex vivo autoradiography studies in Tg-mice and wild-type mice were also performed. The radioactive spots were found to concentrate at the site of the blood vessels in the Tg-mice brain tissue, as identified by in vitro fluorescence staining using thioflavin-S. Biodistribution studies of [**^99m^Tc]116** show a relatively low brain uptake equal to 1.21% ± 0.22% ID/g at 2 min post-injection and rapid blood washout with an approximately 23-fold decline in blood radioactivity at 60 min post-injection. Other complexes showed worse brain uptake. The authors claimed that coordination compounds [**^99m^Tc]109–116** are prospective as cerebrovascular Aβ-visualization agents.

**Figure 28:**
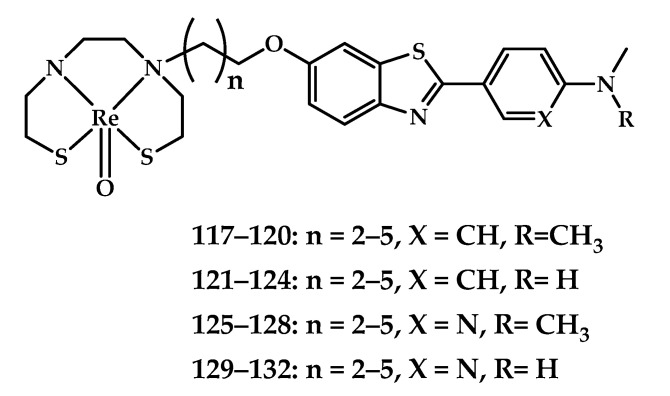
Re(III) coordination compounds **117**–**132** based on 2-arylbenzothiazoles conjugated with a BAT chelating moiety.

Zhang et al. designed a series of sixteen ^99m^Tc-labeled imaging probes [**^99m^Tc]117–132** for Aβ plaques based on 2-arylbenzothiazoles conjugated with a bis(aminoethanethiol) (BAT) chelating moiety and their Re(III) analogues **117**–**132** [90] (Figure 28). An in vitro binding affinity of rhenium complexes **117**–**132** to aggregated Aβ_1__−42_ peptide was estimated by a competitive binding assay using [^125^I]IMPY as a reference ligand. The results obtained proved that both the introduction of a dimethylamine group and an increase in the length of the linker between the amyloid affinity and the metal-chelating moiety promotes Aβ binding of the resulting coordination compounds. Compounds **120** and **122** showed a binding affinity (respectively 8.4 and 8.8 nM) surpassing that of IMPY, a widely used imaging agent. Binding of the coordination compound to Aβ plaques in Tg-mice and AD brain tissue samples was also proven using in vitro fluorescent staining with thioflavin-S as a control.

^99m^Tc-labeled probes [**^99m^Tc]117–132** were obtained using a ligand exchange reaction with ^99m^Tc−glucoheptonate. The ability of the purified ^99m^Tc-labeled probes [**^99m^Tc]118–134** to bind Aβ plaques was tested in brain slices from Tg-mice. Biodistribution studies of ^99m^Tc-labeled complexes were conducted. [**^99m^Tc]124** indicated its suitability as a diagnostic probe. ^99m^Tc-labeled coordination compound [**^99m^Tc]124** showed relatively high initial brain uptake (2.11% ID/g at 2 min) and a reasonable clearance rate (0.62% ID/g at 60 min), in contrast to other complexes, which exhibited poor brain uptake (less than 1% ID/ g at 2 min) and slow clearance, presumably because of their higher lipophilicity and nonspecific binding to plasma proteins.

SPECT images of coordination compound [**^99m^Tc]122** in rhesus monkeys were registered, and the images revealed radioactivity accumulation in the brain, indicating permeation of [**^99m^Tc]121** through the BBB (Table 6). This is the first assessment of a ^99m^Tc-labeled Aβ probe in nonhuman primates.

**Figure 29:**
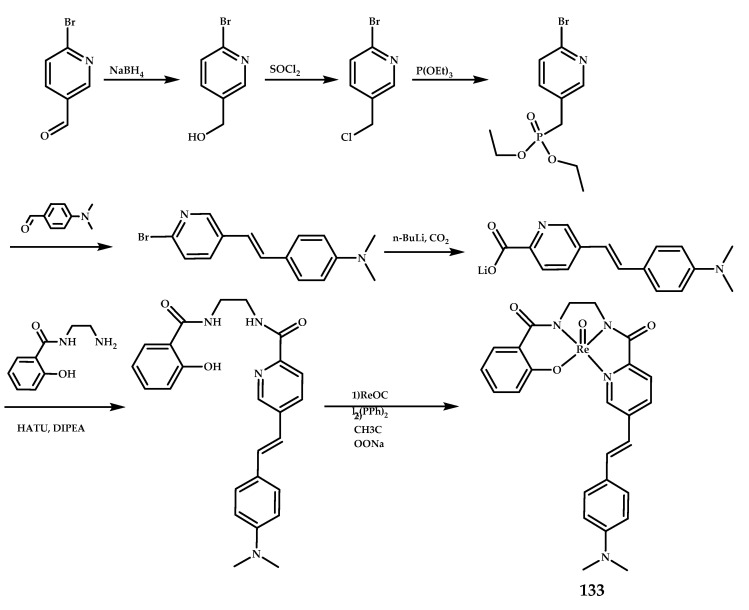
Oxorhenium(V) complexes **133** based on a styrylpyridyl functional group with 2-aminoethyl-2-hydroxybenzamide as a chelating moiety, designed for SPECT imaging of Aβ plaques.

Hayne et al. reported oxotechentium(V) and oxorhenium(V) complexes [**^99m^Tc]133** and **133** based on a styrylpyridyl functional group with 2-aminoethyl-2-hydroxybenzamide as a chelating moiety [91] (Figure 29). The affinity of **133** for Aβ_1−42_ fibrils was estimated to be K_i_ = 855 nM using a fluorescence competition assay against Thioflavin T. It was also shown that **133** binds to Aβ plaques in human brain tissue using human AD brain sections.

**Figure 30:**
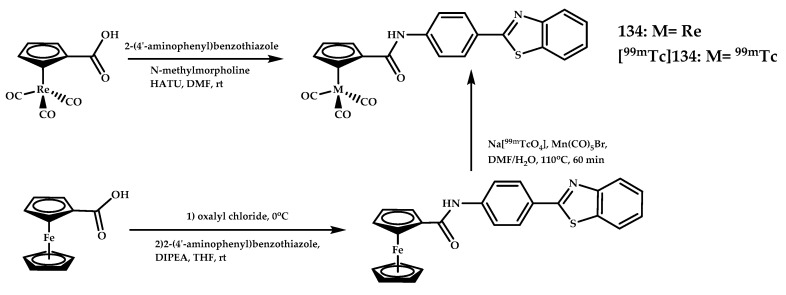
2-(4′-aminophenyl)benzothiazole-based ^99m^Tc-radioagent [**^99m^Tc]134** and its Re(III) analogue **134**, designed for SPECT imaging of Aβ plaques.

Kiritsis et al. reported a 2-(4′-aminophenyl)benzothiazole-based ^99m^Tc-radioagent [**^99m^Tc]134** and its Re(III) analogue **134** [92] (Figure 30). A strong affinity of **134** for Aβ plaques in brain sections from an AD patient was proven using confocal microscopy. The binding affinity of **134** for Aβ_42_ was measured in vitro by competition binding assay between the stable 1**34** and its radioactive ^99m^Tc-labeled analogue [**^99m^Tc]134**, and the obtained K_i_ was 13.6 ± 4.8 nM.

Biodistribution experiments of [**^99m^Tc]134** in Swiss albino mice revealed a moderate initial brain uptake of 0.53% ID/g at 2 min and slow clearance of radioactivity from the brain with a brain_2min_/brain_90min_ ratio of 2.1. Administration of [**^99m^Tc]134** in 5xFAD Tg-mice showed that 0.52% ID/g of radioactivity is recorded in the brain at 2 min, a result similar to that in healthy mice. But the significant increase of radioactivity in the brain of 5xFAD Tg-mice with time (1.94% ID/g at 90 min post-injection) is consistent with retention of [**^99m^Tc]134** through binding to Aβ plaques.

**Figure 31:**
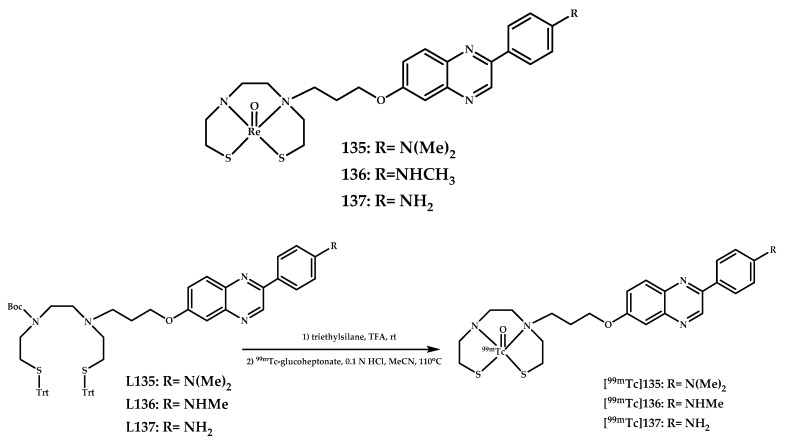
^99m^Tc complexes [**^99m^Tc]135–137** based on a phenylquinoxaline scaffold and their model Re(III) analogues **135**–**137,** designed for SPECT imaging of Aβ plaques.

Iikuni et al. reported three novel ^99m^Tc complexes [**^99m^Tc]135–137** based on a phenylquinoxaline scaffold and their model Re(III) analogues **135**–**137** [93] (Figure 31).

An in vitro binding experiment in solution showed promising Aβ affinity for complex **135** and average binding affinity for complex **136**. The affinity increased in the order of the N,N-dimethylated derivative > N-monomethylated derivative > primary amino derivative.

The brain uptake for ^99m^Tc-labeled complex [**^99m^Tc]135** was found to be 0.88%, and the brain_2min_/brain_60min_ ratio was 3.52. An ex vivo autoradiographic examination was also performed using a Tg2576 mice, and [**^99m^Tc]135** showed intensive radioactive spots in sections from the Tg2576 mice but not from the age-matched mice. In addition, these spots corresponded with Aβ depositions confirmed by fluorescent staining in the same sections with thioflavin-S.

**Figure 32:**
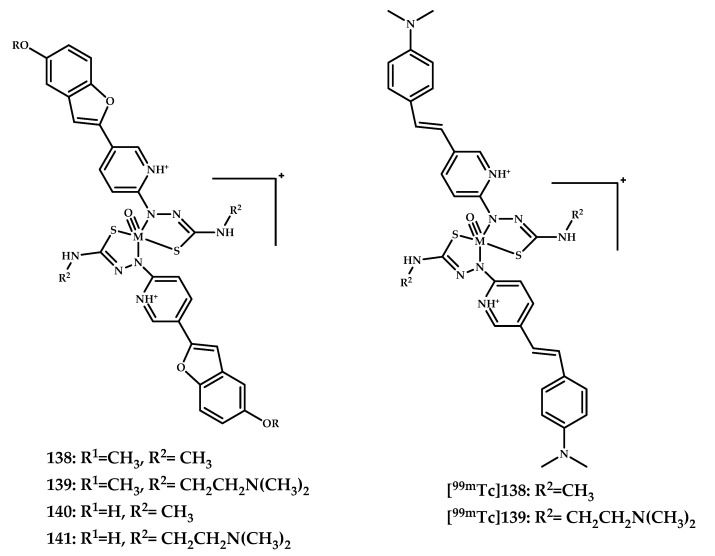
Re(III) complexes **138**–**142** based on styrilpyridyl and benzofuran moieties, and ^99m^Tc labeled coordination compounds [^99m^Tc]**138** and [^99m^Tc]**139,** designed for SPECT imaging of Aβ plaques.

Fletcher et al. reported six Re(III) complexes **138–142** based on styrilpyridyl and benzofuran moieties [94] (Figure 32). An affinity to Aβ plagues was investigated using a ThT assay, and the obtained results suggested that the complexes either bind competitively with ThT to Aβ_1–42_ fibrils or inhibit fibril formation. ^99m^Tc-labeled coordination compounds [**^99m^Tc]138** and [**^99m^Tc]139** were also obtained.

**Figure 33:**
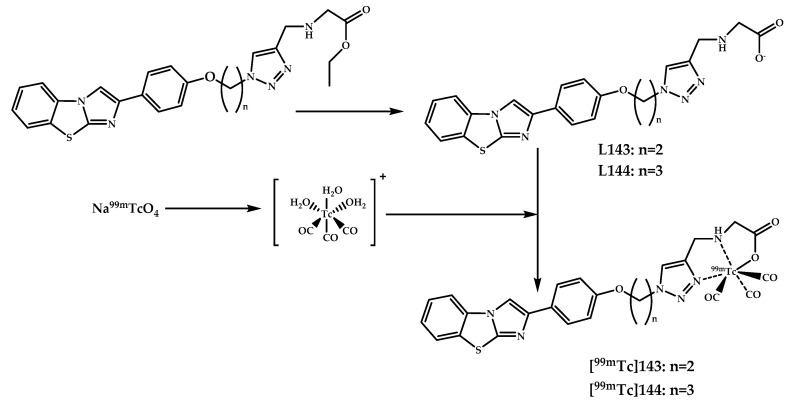
Radiolabeled 2-arylimidazo[2,1-b]benzothiazoles **143** and **144,** designed for SPECT imaging of Aβ plaques.

Molavipordanjani et al. reported two novel radiolabeled 2-arylimidazo[2,1-b]benzothiazoles **143** and **144** [95] (Figure 33). The affinity of the coordination compounds for Aβ_1–42_ aggregates was evaluated, and both radiolabeled complexes showed a significant Aβ binding. Tissue staining and autoradiography with Congo Red as a control proved an ability of the obtained complexes **143** and **144** to bind to Aβ plaques in the brain sections of the rat AD model. Biodistribution studies in normal BALB/C mice showed an initial brain uptake of 0.78% and 0.86% ID/g respectively, for **143** and **144** in normal mice, followed by a nearly complete washout within an hour.

**Figure 34:**
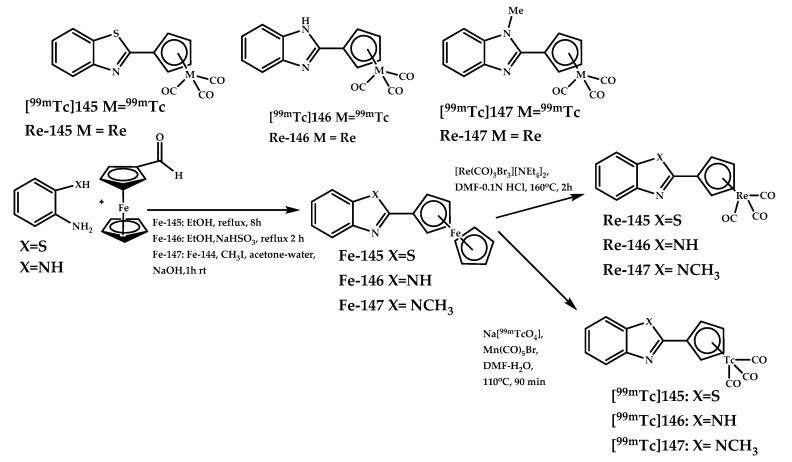
^99m^Tc complexes [**^99m^Tc]145**–[**^99m^Tc]147** and their corresponding Re analogues **145**–**147** designed for SPECT imaging of Aβ plaques.

Sagnou et al. reported synthesis of three novel ^99m^Tc complexes [**^99m^Tc]145**–[**^99m^Tc]147** and their corresponding Re analogues **145**–**147**, in which the phenyl ring of the classical Aβ-binding structures 2-phenylbenzothiazole or 2-phenylbenzimidazole is replaced by cyclopentadienyl tricarbonyl [Cp^99m^Tc(CO)_3_] [96] (Figure 34).

The affinity of complexes **145**–**147** for Aβ plaques was evaluated with confocal microscopy on human AD brain sections. All three complexes bind selectively to the Aβ plaques. Competition binding assays between the stable Re complexes **145**–**147** and their radioactive ^99m^Tc counterparts [**^99m^Tc]145**–[**^99m^Tc]147** showed K_i_ values of 65.8 ± 21.3, 7.0 ± 2.9, and 5.7 ± 2.9 nM. Biodistribution experiments showed brain uptake of [**^99m^Tc]145** (7.94 ± 1.46%) comparable to that of ^18^F-florbetapir (7.33% ID/g at 2 min), fast blood clearance, and lack of retention in brain tissue.

Biodistribution of [**^99m^Tc]145** in 5xFAD Tg-mice showed AD brain accumulation of 3.90 ± 0.19 for Tg-mice and 2.68 ± 0.06 for wild-type mice (15 min post-injection). The Re complexes **145**–**147** also showed an anti-amyloid therapeutic potential.

**Figure 35:**
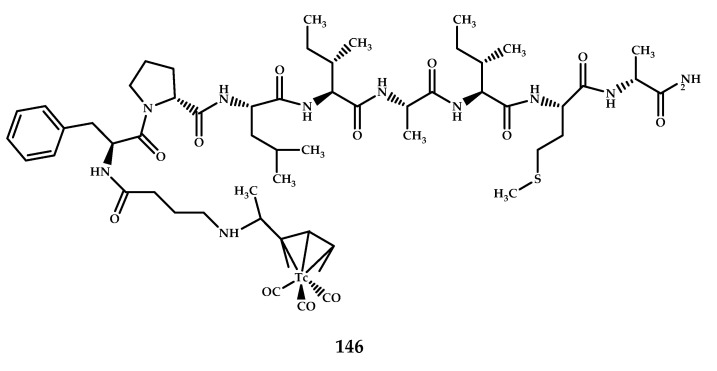
^99m^Tc-Cp-GABA-D-(FPLIAIMA)-NH_2_**148** based on an Aβ-affinitive peptide scaffold, designed for SPECT imaging of Aβ plaques.

Jokar et al. designed a ^99m^Tc agent **148** with a lipophilic peptide scaffold, ^99m^Tc-Cp-GABA-D-(FPLIAIMA)-NH_2_ [97] (Figure 35).

Binding affinity studies were carried out on Aβ aggregation, and the respective observed values of K_d_ and B_max_ were 20.22 ± 7.26 μM and 201,700 ± 8750.89 bound molecules/plaque. In vitro autoradiography studies, scintigraphy, and fluorescence staining were performed on the brain sections of AD and normal rats and also on brain sections of AD, normal, and schizophrenia patients for better confirmation. The radiopeptide displayed a good binding affinity for the Aβ plaques on brain sections of AD rats and a significant binding affinity for Aβ plaques in human brain sections. Brain uptake in AD and normal rats was respectively 0.38% and 0.35%, and brain uptake of radiopeptide on AD brain increased 2 min post-injection and slowly dropped at 30 min, as compared with normal ones. Biodistribution studies in the presence of a p-glycoprotein (PgP) blocker and SPECT/CT imaging studies were also performed following intravenous administration of the probe. The analyzed images showed significant radioactivity uptake in the AD brains compared with uptake in normal rats.

## 5. Conclusions

Among various strategies utilized to obtain copper-based AD imaging agents, compound **1** with a low molecular mass and ATSM chelating moiety demonstrated the highest level of brain uptake at 2 min post-injection. We note that modification of the ATSM moiety with polyamine led to a significant increase in brain uptake. Other Cu-chelating fragments such as DOTA lead to a decrease in brain uptake compared with Cu-ATSM-based complexes.

Gd/Ga complexes designed for MRI and PET imaging of Aβ showed good in vitro activity, but when tested in vivo, those compounds showed little to no BBB penetration, which can result from the presence of rigid DOTA/DO3A, etc., scaffolds used to chelate Gd/Ga. The most potent compound **71** demonstrated a brain uptake of 1.24% ID/g at 2 min post-injection despite a MW ≈ 1000, which is far beyond the optimal mass for BBB penetration.

Some of the ^99m^Tc-based coordination compounds demonstrated promising in vitro and in vivo activity. The most potent complexes for SPECT imaging were compounds **145**–**147** with piano stool moieties coupled with Aβ-binding benzothiazole scaffolds**,** with **145** showing a brain uptake of 7.94% at 2 min post-injection. When rigid chelating structures, long linkers, and heavy Aβ-binding fragments are used, the BBB penetrability of the resulting coordination compounds decreases dramatically, as shown for **92**–**95** and **107**–**132**.

Metal-based imaging agents for AD allow noninvasive imaging of Aβ plaques, a crucial procedure for successful AD diagnosis and therapy. There is a strong need for new efficient AD imaging probes, and this area of research is therefore thriving. The radioisotopes ^64^Cu, ^68^Ga, and ^99m^Tc are promising and can be obtained either by cyclotrons or by radioisotope generators. They also have half-lives much longer than do ^18^F and ^11^C, which are currently used for imaging. Radioactive metal isotopes can be introduced at the last step of synthesizing an imaging agent, which reduces the potential activity loss.

Among the vast variety of compounds considered in this review, the most promising results were shown by Cu^2+^-based coordination compounds **1** and **11** for PET imaging, Gd^3+^-based coordination compound **40** for MRI, and ^99m^Tc-based coordination compound **145** for SPECT imaging, demonstrating the best Aβ-binding affinity and brain uptake at 2 min post-injection while being light-weight complexes with small Aβ-binding fragments.

## Figures and Tables

**Table 1:** Cu(II)-based coordination compounds for positron emission tomography (PET) imagining of Alzheimer disease.

Coordination Compound Number	Brain Uptake, ID/g, 2 min Post-Injection, %	Brain_2min_/_60min_ (*Brain_2min_/_30min_) Ratio	Brain Tissue Experiments	Aβ Binding Moiety	Reference
		Cu(ATSM)-based coordination compounds			
**1**	2.5 ± 0.6 (*APP/PS1* transgenic mice) 1.7 ± 0.6 (Wild-type mice) 7 min after injection	-	Epi-fluorescence microscopy of AD human brain sections	Stilbene	[39]
**2**–**4**	1.11 ± 0.20	2.92 *	Epi-fluorescence microscopy of AD human brain sections	2-benzothiazole,3,4-styrylpyridine	[40]
**5**–**8**	1.39 ± 0.06 1.06 ± 0.43 0.77 ± 0.19 1.54 ± 0.60	1.31 * 2.16 * 1.05 *	Elemental composition of AD human brain tissue using LA-ICP-MS	Benzofuran	[41]
**9**	-	-	Epi-fluorescence microscopy of AD human brain tissue (ligand)	Stilbene	[42]
**10**–**15**	2.2 ± 0.6 1.1 ± 0.2	6.47 5	Epi-fluorescence microscopy of AD human brain sections	Styrylpyridine	[43]
**16**–**25**	4.41 ± 0.23 (23 h Post-injection similar)	-	PET imagine of BALB/c mice	-	[44]
Other metal-chelating moieties					
**26, 27**	0.33 ± 0.12 0.36 ± 0.10	1.83 2.11	Fluorescent staining using brain sections from a Tg2576 mice	Benzofuran	[45]
**29**–**33**	0.37 ± 0.06 0.17 ± 0.02 1.33 ± 0.27 0.49 ± 0.01 0.61 ± 0.14 0.75 ± 0.16	2.64 1.30 4.92 2.22 4.69 2.88	Fluorescent imaging of amyloid plaques in Tg2576 AD mice brain sections	Benzothiazole	[46,47]
**34**–**39**	0.16 ± 0.02 0.99 ± 0.04	1.59 4.95	Fluorescence imaging of amyloid plaques in 5xFAD mice brain sections	Benzothiazole	[48]

* Brain_2min_/_30min_ ratio is indicated instead of Brain_2min_/_60min_ ratio.

**Table 2:** Gd^3+^, Ga^3+^ coordination compounds for magnetic resonance imaging (MRI) and single-photon emission computed tomography (SPECT) imaging of AD.

№	Brain Uptake, %	Diagnostic Method	Metal	Metal- Chelating Moiety	Aβ-binding Moiety	Reference
**40**–**42**	Cellebrium 0.50 ± 0.07 Cortex 0.36 ± 0.03	MRI SPECT	Gd^3+^, ^111^In^3+^	DO3A	PiB	[55]
	-					
**43, 44**	-	MRI	Gd^3+^	DO3A	PiB	[56]
**45**–**60**		MRI	Gd^3+^	DOTA PCTA	Benzothiazole Benzoxazole Stilbene	[57]
**61**	-	PET	Ga^3+^	DOTA	Benzofuran	[58]
**62**–**64**	-	PET	Ga^3+^	DOTA	PiB	[59]
**65**–**70**	0.12 ± 0.05 0.17 ± 0.05 0.31 ± 0.09 0.21 ± 0.05 0.22 ± 0.03 0.11 ± 0.01	PET	Ga^3+^	HBED-CC	Styrylpyridine	[60]
**71**	1.24 ± 0.31	PET	Ga^3+^	Chalkone		[61]
**72** **74**	No brain uptake	-	Ga^3+^	Curcumin		[62,63]
**75**	No biodistribution experiment	-	Ga_3+_	N_2_O_2_ Schiff- base ligand	Curcumin	[64]
**76, 77**		-	Ga^3+^	NODAGA AAZTA	Curcumin	[65]
**78**–**88**	0.21 ± 0.07 (5 min p.i.)	PET SPECT	Ga^3+^ ^99m^Tc^3+^	DOTA	Tacrine	[66]

**Table 3:** Log D values for coordination compounds **78**–**88**.

(CH_2_)_n_	Log D		
	[^99m^Tc]Tc-Hynic-NH(CH_2_)_n_Tac		[^68^Ga]Ga-DOTA-NH(CH_2_)_n_Tac
**78**: *n* = 2	−2.95 ± 0.06		-
**79**: *n* = 3	−2.80 ± 0.01		-
**80**: *n* = 4	−2.53 ± 0.02		-
**81**: *n* = 5	−2.41 ± 0.01		-
**82**: *n* = 6	−2.08 ± 0.01		-
**83**: *n* = 7	−1.86 ± 0.02	**86**:	−2.52 ± 0.01
**84**: *n* = 8	−1.50 ± 0.01	**87**:	−2.02 ± 0.01
**85**: *n* = 9	−1.38 ± 0.01	**88**:	−1.52 ± 0.01

**Table 4:** The activity of **82** and **86** against two cholinesterases.

Compound	IC_50_ ± SD ** (nM)		Selectivity for AChE ^a^	Selectivity for BuChE ^b^
	AChE	BuChE		
**82**	0.10 ± 0.01	0.12 ± 0.02	1.2	0.83
**86**	290 ± 20	167 ± 9	0.57	1.75
Tacrine	107 ± 9	16 ± 1	0.15	6.67

^a^ Selectivity for AChE is defined as IC_50_(BuChE)/IC_50_(AChE); ^b^ Selectivity for BuChE is defined as IC_50_(AChE)/IC_50_(BuChE). ** half maximal inhibitory concentrations ± standard deviation

**Table 5:** ^99m^Tc coordination compounds for single-photon emission computed tomography (SPECT) visualization of AD.

№	Brain Uptake, ID/g, 2 Min Post-Injection %	Brain_2 min_/Brain_60 min_ Ratio	Brain Tissue Experiments	Ligand	Reference
**89**–**91**	4.10 ± 0.38 /6.34 ± 0.81 2.30 ± 0.27 /3.68 ± 0.07 1.11 ± 0.34/ 1.64 ± 0.17 With/without PgP Blocked by Cyclosporin A	8.20 4.18 1.73	Fluorescent staining of Re complexes on *APPswe/PSEN1* mice and AD patient brain sections Autoradiography on a *APPswe/PSEN1* model mice	Chalcone-mimic moiety with [Cp^99m^Tc(CO)_3_]	[83]
**92**–**95**	0.49 ± 0.08 0.47 ± 0.11 0.48 ± 0.06 0.31 ± 0.06	6.13 3.92 5.33 2.06	In vitro fluorescent staining of Re complexes of brain tissue *APPswe/PSEN1* mice	Curcumin-like dibenzylideneacetone conjugated with monoamineemonoamide dithiol (MAMA) and BAT (bis(aminoethanethiol) as chelating moieties	[84]
**96**–**100**	0.28 ± 0.03	2.54	Autoradiography *Tg2576* and wild-type mice	Benzotiasole/stilbene conjugated with hydroxamamide (Ham) as chelating moiety	[85,86]
**101**–**104**	0.25 ± 0.05 0.24 ± 0.02 (wild type/*APP* mice)	1.26	SPECT images in *APP/ PS1* transgenic mice	Styrilpyridyl conjugated with pyridylamine-carboxylate and dipyridylamine ligands as chelating moiety	[87]
**105**–**107**	1.10 ± 0.08 0.96 ± 0.13 1.55 ± 0.51 1.24 ± 0.17	3.54 6.40 3.87 8.64	In vitro autoradiography Brain tissue from *APPswe/PSEN1* mice	Arylbenzoxazole conjugated with bis (aminoethanethiol) (BAT) as chelating moiety	[88]
**109**–**116**	0.80 ± 0.17 0.61 ± 0.08 0.88 ± 0.14 1.21 ± 0.22	26.66 3.38 6.68 20.16	Fluorescent staining of Re complexes with brain sections of *APPswe/PSEN1T* mice and AD patients In vitro autoradiography on brain sections of *APPswe/PSEN1T* mice and AD patients	Benzothiazole conjugated with iminodiacetic acid (IDA) as chelating moiety	[89]
**117**–**132**	0.69 ± 0.16 0.46 ± 0.09 0.59 ± 0.12 2.11 ± 0.11 0.92 ± 0.09 0.47 ± 0.07 0.60 ± 0.05	1.50 1.15 1.37 3.40 1.46 2.47 2.07	Fluorescent staining of rhenium complexes on brain slices from *APPswe/PSEN1* mice and AD patients. Autoradiography on brain slices from *APPswe/PSEN1* mice Ex vivo Autoradiography *APPswe/PSEN1* mice In vivo SPECT−CT Imaging in Rhesus Monkeys	Arylbenzoxazole conjugated with bis (aminoethanethiol) (BAT) as chelating moiety	[90]
**131**	-	-	Fluorescent staining or De complexes of AD human brain tissue	Styrilpyridyl conjugated with 2-aminoethyl-2-hydroxybenzamide as chelating moiety	[91]
**132**	0.53 ± 0.11 0.52 ± 0.08 (healthy/5xFAD mice)	Brain_2 min_/Brain_90 min_ 2.0 Brain _2 min_/Brain _90min_ 2.1.	Fluorescence staining of Re complexes of AD patient brain and 5x FAD mice	Benzothiazole conjugated with tricarbonyl [M(CO)_3_]^+^	[92]
**133**–**135**	0.88± 0.08	3.52	Ex vivo autoradiography using Tg2576 mice	Phenylquinoxaline conjugated with bis (aminoethanethiol) (BAT) as chelating moiety	[93]
**136**–**140**	_	-	Fluorescence staining of Re complexes of AD patient brain	Styrilpyridyl/Benzofuran conjugated with pyridylthiosemicarbazide as chelating moiety	[94]
**141, 142**	0.78 ± 0.07 0.86 ± 0.07	8.66 7.16	Autoradiography of AD rat model (vaccinated with Aβ solution)	Arylimidazo[2,1-b] benzothiazole conjugated with triazole-based N/N/O, N/N/N, N/N/S ligands as chelating moieties	[95]
**143**–**145**	7.94 ± 1.46 3.99 ± 0.60 5.36 ± 0.65	39.7 99.75 59.55	Fluorescent staining of AD patient brain	Benzothiazole with benzene ring replaced by the cyclopentadienyl tricarbonyl	[96]
**146**	0.38 ± 0.03 0.35 ± 0.01 (AD/normal rats) With/without blocked PgP (Cyclosporine A) (10 min after injection) 0.27 ± 0.01 0.60 ± 0.01	Brain2 min/brain30 min 2.33 Brain2 min/brain30 min 1.65	Planar scintigraphy, autoradiography and fluorescent staining with Thioflavin S and Congo Red studies on prepared brain slices of AD rats (vaccinated with Aβ1–42) and brain sections of AD and Schizophrenia patients.	D-(FPLIAIMA)-NH_2_ peptide	[97]

**Table 6:** **[^99m^Tc]122** brain accumulation in rhesus monkeys (M04: 4-year-old, male; F27: 27-year-old, female).

	0–10 Min	10–20 Min	20–30 Min	30–40 Min	Clearance Ratio
M04	1.23	1.13	1.01	0.88	1.40
F27	0.78	0.70	0.67	0.64	1.22
